# Preliminary Results: The Impact of Smartphone Use and Short-Wavelength Light during the Evening on Circadian Rhythm, Sleep and Alertness

**DOI:** 10.3390/clockssleep3010005

**Published:** 2021-01-22

**Authors:** Christopher Höhn, Sarah R. Schmid, Christina P. Plamberger, Kathrin Bothe, Monika Angerer, Georg Gruber, Belinda Pletzer, Kerstin Hoedlmoser

**Affiliations:** 1 Laboratory for Sleep, Cognition and Consciousness Research, Department of Psychology, University of Salzburg, 5020 Salzburg, Austria; christopher.hoehn@sbg.ac.at (C.H.); sarah.schmid@stud.sbg.ac.at (S.R.S.); christina.plamberger@sbg.ac.at (C.P.P.); kathrin.bothe@sbg.ac.at (K.B.); monika.angerer@sbg.ac.at (M.A.); 2 Centre for Cognitive Neuroscience Salzburg (CCNS), University of Salzburg, 5020 Salzburg, Austria; Belinda.Pletzer@sbg.ac.at; 3 The Siesta Group, 1210 Vienna, Austria; georg.gruber@thesiestagroup.com

**Keywords:** LED-screens, light exposure, short-wavelength light, blue light filter, sleepiness, slow wave sleep, slow wave activity, melatonin, cortisol, skin temperature

## Abstract

Smartphone usage strongly increased in the last decade, especially before bedtime. There is growing evidence that short-wavelength light affects hormonal secretion, thermoregulation, sleep and alertness. Whether blue light filters can attenuate these negative effects is still not clear. Therefore, here, we present preliminary data of 14 male participants (21.93 ± 2.17 years), who spent three nights in the sleep laboratory, reading 90 min either on a smartphone (1) with or (2) without a blue light filter, or (3) on printed material before bedtime. Subjective sleepiness was decreased during reading on a smartphone, but no effects were present on evening objective alertness in a GO/NOGO task. Cortisol was elevated in the morning after reading on the smartphone without a filter, which resulted in a reduced cortisol awakening response. Evening melatonin and nightly vasodilation (i.e., distal-proximal skin temperature gradient) were increased after reading on printed material. Early slow wave sleep/activity and objective alertness in the morning were only reduced after reading without a filter. These results indicate that short-wavelength light affects not only circadian rhythm and evening sleepiness but causes further effects on sleep physiology and alertness in the morning. Using a blue light filter in the evening partially reduces these negative effects.

## 1. Introduction

The use of electronic media (e.g., smartphones) in the evening rises continuously [1], especially among adolescents and young adults [2]. Smartphones, laptops and e-books are equipped with light-emitting diodes (LEDs), which emit a considerable amount of short-wavelength light (i.e., “blue light”) [3,4]. These light sources show a spectral peak around 460 nm [5,6]. There is growing evidence that high amounts of short-wavelength light, as well as bright light in general [7], during the evening can reduce subjective and objective levels of sleepiness and increase alertness. It has to be noted, that these terms—sleepiness and alertness—are often used interchangeably in the field [5,8,9,10]. For instance, one study reported a delayed sleep onset and reduced sleepiness after reading for 2 h in an e-book emitting high portions of short-wavelength light [11]. Similar results were obtained in another study which demonstrated that intermittent blue-enriched light was as effective as constant blue-enriched light in reducing sleepiness in the evening and shortening subsequent sleep time as well as sleep efficiency [12]. A delayed sleep onset but constant wake-up times (e.g., due to fixed work schedules) shorten the total sleep duration and thereby lead to a phenomenon called “social jetlag”. This term describes a dissonance between the biological and the social clock and is associated with an increased risk for psychological and also somatic diseases like obesity [13]. Interestingly, blue light blocking glasses have been shown to reduce the alerting effects of blue light in the evening [14] and therefore may act as a countermeasure.

When light reaches the retina, it gets not only absorbed by classical retinal photoreceptor cells (i.e., rods and cones) but also by specific intrinsically photosensitive retinal ganglion cells (ipRGCs) [15], which contain the photopigment melanopsin [16,17]. Melanopsin is especially sensitive to light with short wavelengths between 446 nm and 480 nm [18], thus to light emitted preferentially by LED screens (e.g., smartphones). Information about light is further transported via the retinohypothalamic tract to our “inner clock” located in the suprachiasmatic nuclei (SCN) of the hypothalamus. The SCN then project to the pineal and pituitary glands. The pituitary gland controls and stimulates the secretion of the stress hormone cortisol [19,20]. The cortisol secretion shows a circadian course, characterized by a declining concentration during the day, and is reaching a nadir (i.e., lowest concentration) around midnight [21]. In the morning, cortisol concentration steeply increases within 30 min to 60 min after awakening (i.e., cortisol awakening response (CAR)) [21]. The CAR reflects the hypothalamus-pituitary-adrenal (HPA) axis activity in response to the transition from sleeping to waking state and is also influenced by the circadian timing [22]. Early awakenings have been associated with a stronger CAR [23]. One study showed that greater CAR responses in the morning may be driven by N2 sleep, especially those N2 periods preceding slow wave sleep (SWS, i.e., N3 sleep) [24]. Regarding light-induced cortisol changes, exposure to blue-enriched light (1500 lux) in the evening (23:00 to 24:00) did not show an immediate effect on cortisol secretion [25], whereas morning (05:00 to 08:00) bright light exposure (2000 to 4500 lux) elevated cortisol secretion [26]. However, outlasting effects of blue-enriched or bright light exposure in the evening on cortisol levels in the morning were not assessed in these studies.

In addition to cortisol, the secretion of the sleep “facilitating” hormone melatonin also follows a circadian rhythm, which is controlled by the pineal gland, feeding back to the SCN [27]. Thereby melatonin reinforces the sleep pressure and inhibits the propensity to stay awake [27,28]. Regarding the impact of light, melatonin is strongly affected by short-wavelength light exposure in the evening. For instance, 2 h of 460 nm light exposure in the late evening (i.e., 21:30 to 23:30) compared to 540 nm or no light suppressed the melatonin secretion significantly [29]. In line with these results, 5 h of LED-screen exposure preceding sleep initially suppressed melatonin secretion, followed by a delayed increase [5]. Interestingly, wearing blue light blocking glasses while sitting in front of a LED-screen attenuated the induced melatonin suppression [14]. These results indicate a pronounced impact of blue-enriched light on melatonin secretion before sleep. In general, melatonin is commonly known as the “sleep hormone“ but should be better considered as a mediator between the SCN-activity and thermoregulation. Indeed, the evening rise in melatonin secretion precedes a cascade of thermoregulatory changes: while skin temperature at distal parts of the body (e.g., feet, hands) rises, skin temperature at proximal parts of the body (e.g., subclavicular region) and core body temperature (CBT) decline, indicating a decrease in heat production and an increase in heat loss (i.e., due to increased distal vasodilation) during the evening [30]. An indirect measure for this heat loss is the distal-proximal gradient (DPG, i.e., difference between skin temperature at distal and proximal locations), which has also been shown to be associated with subjective sleepiness [31] and sleep onset latency [32]. In the morning, melatonin secretion regresses to baseline level whereas CBT increases steeply [33,34]. Earlier studies assessed the influence of light exposure on thermoregulatory mechanisms. After 3 h of short-wavelength light exposure CBT remained higher during the first half of the night. This was not the case after dim light exposure [35]. Moreover, the DPG decreased after 2 h of light exposure to wavelengths of 460 nm and 550 nm [29]. Regarding the relationship between DPG and sleep, a physical exercise-induced increase in DPG during the subsequent night was identified as a positive predictor for increased SWS [36].

This allows the assumption that evening light exposure might also have an impact on sleep physiology, especially on the amount of SWS and on slow wave activity (SWA, 0.75–4.5 Hz), respectively. Indeed, two studies reported reduced SWS and SWA within the first sleep cycle after participants were exposed to short-wavelength light during the late evening (i.e., between 21:30 and 23:30) [6,37]. Similarly, another study showed that reading from a tablet instead of reading a printed book for 30 min before bedtime reduced SWA selectively during the first 2 h of sleep [38]. Even though a rebound effect with an increase in SWA during the third sleep cycle was reported in one study [6]. These findings suggest that especially early sleep is affected by evening short-wavelength light exposure. However, the negative effects on early sleep may be strongly dependent on specific characteristics of the light sources and therefore vary considerably across studies [39]. Thus, the robustness of short-wavelength light effects on sleep may be affected by different study designs and further research is needed to clarify the necessary circumstances for these effects to be detectable. In addition, light-induced negative effects on sleep may impact next-morning behavior. Evidence from earlier studies shows inverse result patterns for alertness and sleepiness in the morning after sleep [9,11,40,41,42]. Thus, short-wavelength light on the preceding evening reduced next-morning alertness because its alerting effects in the evening may have interfered with the subsequent sleep.

An additional interesting question is whether blue light filtering software, which suppresses the amount of emitted short-wavelength light, is suited to reduce the effects of evening light exposure on circadian rhythm, sleep and alertness. While some studies were able to show that blue light filtering software can indeed reduce some of the negative effects of evening short-wavelength light [43,44,45], others failed to show consistent beneficial effects [46], or suggested additional interfering factors such as display brightness [47]. Thus, the research on the usefulness of blue light filters is still somewhat limited and needs further investigation. Therefore, we here not only aimed at investigating the effects of evening short-wavelength light from a smartphone screen but also assessed beneficial effects of the application of a blue light filter. Our study was conducted in a standardized environment under realistic conditions without any additional dark adaptation or an artificially prolonged exposure duration. We present preliminary results of the first 14 participants from a within-subject design in which we assessed the impact of reading for a duration of 90 min during the evening. Subjects either read on a smartphone without (condition: “No Filter”) or with (condition: “Filter”) a blue light filter or on printed material (condition: “Book”). We expected that reading without a blue light filter would lead to reduced sleepiness and melatonin secretion during the evening as well as to a lower DPG during the night and a reduced CAR in the next morning. Additionally, early slow wave sleep (SWS and SWA during the first night-quarter) was expected to be diminished and alertness levels in the next morning were predicted to be reduced. These effects should have been more pronounced when reading on a smartphone without a blue light filter compared to reading with the filter switched on. The reading condition on printed material served as a control condition. In the blue light filter condition the effects should have been diminished due to a reduced amount of short-wavelength light.

## 2. Results

Results are considered statistically significant with an error probability of *p* ≤ 0.05 (*) and all results with *p* ≤ 0.10 (°) are referred to as a statistical trend. Follow-up tests which were no longer significant after Bonferroni correction for multiple comparisons (i.e., *p* > 0.10) are indicated with a cross (†), otherwise the adjusted *p*-values are reported.

### 2.1. Subjective Sleepiness

The ratings on the Karolinska Sleepiness Scale (KSS; [48]) indicated a similar pattern for all light conditions with a general increase in sleepiness over the evening and a rapid decline during the next morning. A statistical trend after 30 min of reading in the evening (21:08) indicated a difference between the three reading conditions (*χ*^2^(2) = 4.92; *p* = 0.086; *W* = 0.18). Follow-up tests showed that sleepiness ratings were higher after 30 min of reading in the “Book” condition as compared to reading in the “No Filter” (*z*(*N* = 14) = 2.00; *p* = 0.046 ^†^; *r* = 0.53) or “Filter” (*z*(*N* = 14) = 1.93; *p* = 0.053 ^†^; *r* = 0.52) condition.

**Figure 1:**
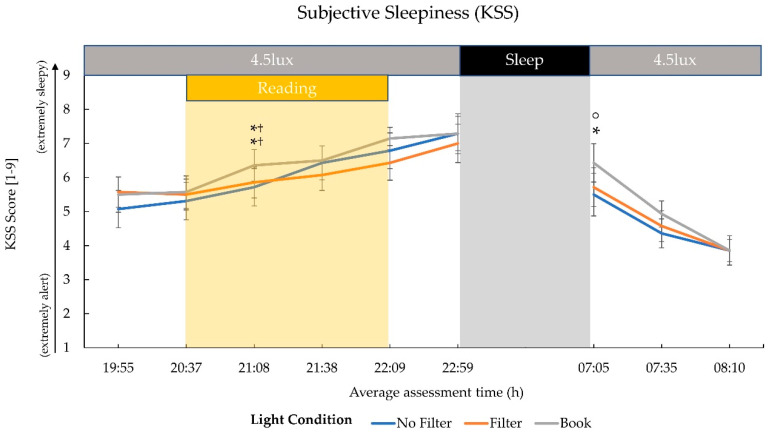
Trajectory of subjective sleepiness ratings (mean and standard error) on the Karolinska Sleepiness Scale (KSS). Administration times averaged over all participants and conditions at the corresponding assessment are shown on the *x*-axis. The 4.5 lux label corresponds to the background room lighting and refers to photopic lux. During the 8 h sleep interval, lights were switched off (0 lux). Subjective sleepiness was reduced in both smartphone conditions compared to the book condition at 21:08 and 07:05. ° = *p* ≤ 0.10; * = *p* ≤ 0.05; ^†^ = *p*_adj_. > 0.10.

Another significant difference between light conditions emerged immediately after awakening (*χ*^2^(2) = 8.22; *p* = 0.016; *W* = 0.29). Here, the “Book” condition again revealed by trend a higher level of sleepiness compared to the “No Filter” (*z*(*N* = 14) = 2.28; *p_adj._* = 0.069; *r* = 0.60) and significantly higher compared to the “Filter” (*z*(*N* = 14) = 2.49; *p_adj._* = 0.039; *r* = 0.68) condition (cf., Figure 1).

### 2.2. Cortisol and Melatonin

**Figure 2:**
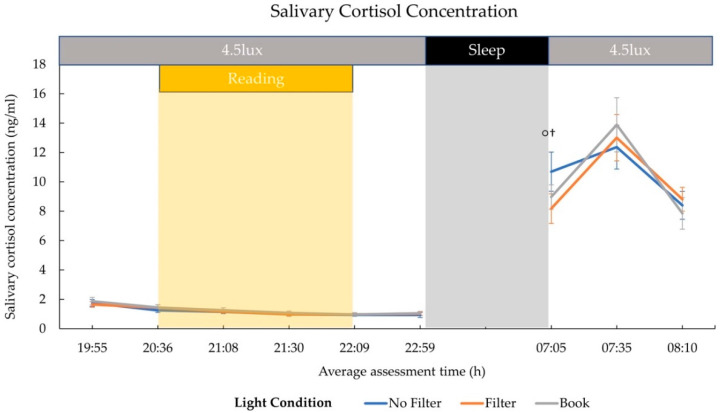
Time course of salivary cortisol concentration (mean and standard error). The 4.5 lux label corresponds to the background room lighting and refers to photopic lux. During the 8 h sleep interval, lights were switched off (0 lux). Cortisol concentration at awakening was by trend higher in the “No Filter” than in the “Filter” condition. Another statistical trend was present for a higher cortisol increase in the “Filter” compared to the “No Filter” condition within 30 min after awakening (cf., Supplementary Figure S1). ° = *p* ≤ 0.10; ^†^ = *p*_adj_. > 0.10.

Salivary cortisol concentration did not differ significantly at awakening between all conditions in general (*F*(2,24) = 1.82; *p* = 0.184; *η*^2^ = 0.13). However, explorative post-hoc comparisons indicated by trend a higher cortisol level at awakening in the “No Filter” than in the “Filter” (*t*(12) = 2.11; *p* = 0.056 ^†^; *d* = 0.59) condition (cf., Figure 2). Further, the increase in the cortisol concentration from awakening to 30 min after awakening did not differ significantly between all conditions (*F*(1.40,16.85) = 1.86; *p* = 0.191; *η*^2^ = 0.13). Again, explorative post-hoc comparisons indicated a statistical trend for a reduced cortisol increase in the “No Filter” compared to the “Filter” condition (*t*(12) = −2.76; *p_adj._* = 0.052; *d* = 0.77). The cortisol concentration increased on average by 15.68% (*SD* = 58.27%) in the “No Filter” condition and by 59.00% (*SD* = 74.12%) in the “Filter” condition (cf., Supplementary Figure S1).

**Figure 3:**
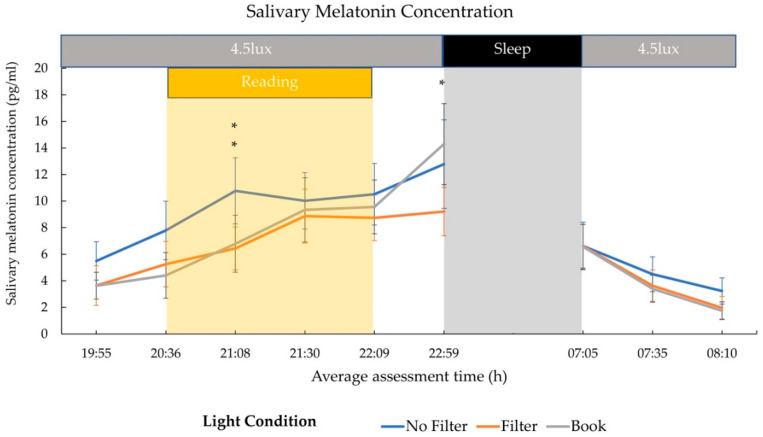
Trajectory of salivary melatonin concentration (mean and standard error). The 4.5 lux label corresponds to the background room lighting and refers to photopic lux. During the 8 h sleep interval, lights were switched off (0 lux). Melatonin concentration was higher in the “No Filter” condition than in both other conditions after 30 min of light exposure and higher in the “Book” condition compared to the “Filter” condition immediately before sleep. * = *p* ≤ 0.05.

Salivary melatonin concentration differed significantly after 30 min of light exposure (21:08) between conditions (*F*(2,26) = 6.29; *p* = 0.006; *η*^2^ = 0.33). Post-hoc comparisons showed that the melatonin concentration was significantly higher in the “No Filter” compared to the “Filter” (*t*(13) = 3.16; *p_adj__._* = 0.023; *d* = 0.84) condition and in the “No Filter” compared to the “Book” condition (*t*(13) = 3.54; *p_adj._* = 0.011; *d* = 0.95) after 30 min of light exposure. Furthermore, salivary melatonin concentration differed by trend between conditions before lights were turned off (22:59; *F*(2,26) = 3.06; *p* = 0.064; *η*^2^ = 0.19). Post-hoc comparisons indicated that salivary melatonin was higher in the “Book” than in the “Filter” (*t*(13) = 2.92; *p_adj._* = 0.036; *d* = 0.78) condition before sleep (cf., Figure 3). Because of descriptively existing a priori differences (statistical not significant), additional baseline corrected values were computed by calculating the change values using the melatonin concentration immediately before the light exposure (20:36) as a baseline. The main effect of light condition after 30 min of light exposure vanished in the baseline-corrected analysis (*F*(2,26) = 2.27; *p* = 0.124; *η*^2^ = 0.15). However, explorative follow-up comparisons still indicated a trend for a higher melatonin concentration in the “No Filter” compared to the “Filter” (*t*(13) = 2.09; *p* = 0.057 ^†^; *d* = 0.56) condition. Before sleep, a significant main effect for light condition was also present in the baseline corrected data (*F*(2,26) = 3.74; *p* = 0.037; *η*^2^ = 0.22). Post-hoc comparisons indicated that the melatonin level was higher in the “Book” than in the “Filter” (*t*(13) = 3.28; *p_adj._* = 0.018; *d* = 0.88) condition (cf., Supplementary Figure S2).

### 2.3. Body Temperature

**Figure 4:**
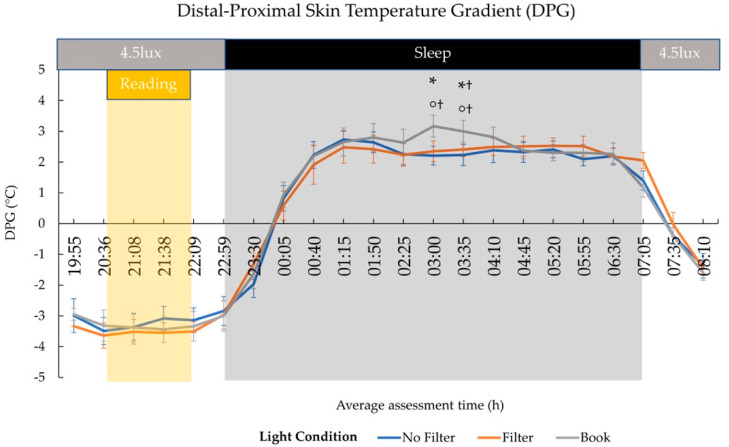
Time Course of Distal-Proximal Skin Temperature Gradient (DPG) values (mean and standard error). The 4.5 lux label corresponds to the background room lighting and refers to photopic lux. During the 8 h sleep interval, lights were switched off (0 lux). DPG was higher in the “Book” compared to the “No Filter” condition and by trend higher in the “Book” than in the “Filter” condition around 03:00 and 03:35 during the night. Values are averaged around the depicted time points ±15 min, which refer to the administration points of the KSS and saliva samplings during the evening and morning. * = *p* ≤ 0.05; ° = *p* ≤ 0.10; ^†^ = *p_adj_*_._ > 0.10.

A statistical trend at 03:00 (*F*(2,22) = 3.30; *p* = 0.056; *η*^2^ = 0.23) and at 03:35 (*F*(2,22) = 3.30; *p* = 0.106; *η*^2^ = 0.18) indicated differences in the DPG between light conditions. Post-hoc comparisons showed a significantly higher DPG at 03:00 in the “Book” than in the “No Filter” condition (*t*(11) = 2.70; *p* = 0.021; *d* = 0.78) and a trend for a higher DPG in the “Book” than in the “Filter” (*t*(11) = 2.08; *p* = 0.062 ^†^; *d* = 0.60) condition. Furthermore, pairwise comparisons revealed a higher DPG at 03:35 in the “Book” condition compared to the “No Filter” (*t*(11) = 2.41; *p* = 0.035 ^†^; *d* = 0.70) condition and a trend for a higher DPG in the “Book” than in the “Filter” (*t*(11) = 1.94; *p* = 0.079 ^†^; *d* = 0.56) condition (cf., Figure 4).

### 2.4. Sleep Measures

Because of technical issues (the recording crashed during the night) one participant was excluded from all sleep analyses. An overview on the general sleep architecture of the participants across the three experimental nights is given in Table 1.

An additional explorative analysis on the sleep fragmentation index (defined as the number of awakenings within the total sleep period per hour sleep) revealed a statistical trend for the main effect of light condition (*χ*^2^(2) = 5.88; *p* = 0.053; *W* = 0.23). Post-hoc tests showed that the sleep was more fragmented over the whole night in the “No Filter” than in the “Book” condition (*z*(*N* = 13) = 2.27; *p_adj._* = 0.069; *r* = 0.63) and also in the “Filter” compared to the “Book” condition (*z*(*N* = 13) = 1.77; *p* = 0.077 ^†^; *r* = 0.49).

**Figure 5:**
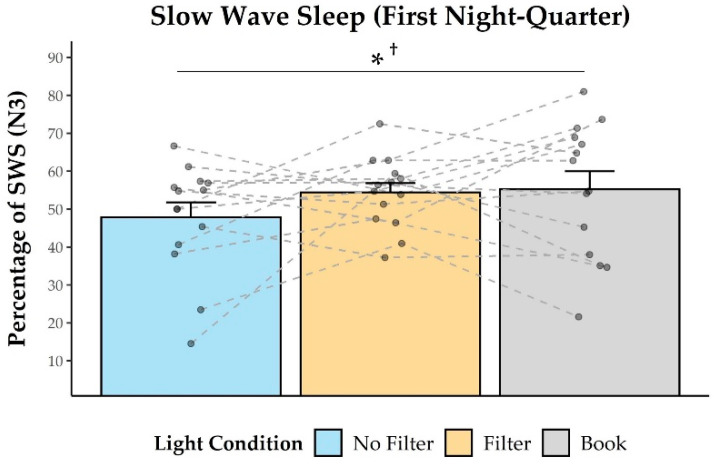
Time in slow wave sleep (N3; in %) during the first night-quarter. Participants spent less time in SWS in the “No Filter” than in the “Book” condition in the first night-quarter. * = *p* ≤ 0.05; ^†^ = *p*_adj._ > 0.10.

Time in SWS during the first night-quarter closely failed to show a statistical trend for the main effect of light condition (*F*(2,24) = 2.48; *p* = 0.105; *η*^2^ = 0.17). Explorative post-hoc comparisons indicated that subjects spent less time in SWS in the “No Filter” than in the “Book” (*t*(12) = −2.40; *p* = 0.034^†^; *d* = 0.67) condition (cf., Figure 5). These effects were restricted to the first night quarter and were not evident when analyzing SWS time during the whole night (*F*(2,24) = 1.18; *p* = 0.324; *η*^2^ = 0.09; cf., Supplementary Figure S3).

**Figure 6:**
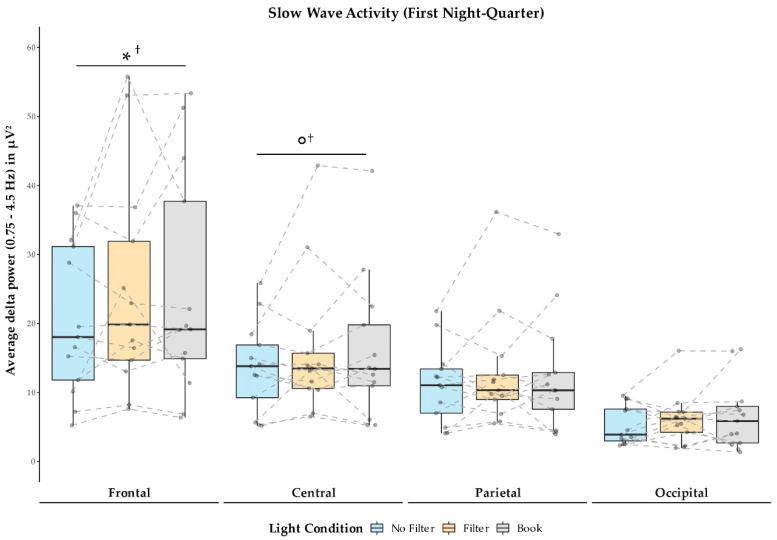
Average SWA (delta power between 0.75 and 4.5 Hz) during N2 and N3 sleep in the first night-quarter across electrode positions and light conditions. SWA was reduced in the “No Filter” compared to the “Book” condition on frontal and central positions. ° = *p* ≤ 0.10; * = *p* ≤ 0.05; ^†^ = *p*_adj._ > 0.10.

In general, SWA was descriptively highest at frontal sites and declined anterior to posterior during the first night-quarter (cf., Figure 6). Even though the main effects for light condition on the different electrode positions did not reach significance (*p* > 0.368), explorative follow-up comparisons indicated that SWA was reduced in the “No Filter” compared to the “Book” condition at frontal (*z*(*N* = 13) = −2.06; *p* = 0.039 ^†^; *r* = 0.57) and central (*z*(*N* = 13) = −1.78; *p* = 0.075 ^†^; *r* = 0.49) electrodes. This SWA reducing effect of short-wavelength light in the “No Filter” condition during the first night-quarter vanished throughout the night (cf., Supplementary Figures S4–S6).

It should be noted that an additional effect of light condition on SWA during the whole night emerged (cf., Supplementary Figure S7). A statistical trend for the main effect of light condition was present over frontal derivations (*χ*^2^(2) = 4.77; *p* = 0.092; *W* = 0.18), whereas SWA at all other electrode positions did not indicate any main effects (*p* > 0.199). Similar to the first night-quarter, pairwise comparisons revealed a reduction of frontal SWA in the “No Filter” compared to the “Book” condition (*z*(*N* = 13) = −1.78; *p* = 0.075 ^†^; *r* = 0.49). SWA was also reduced in the “No Filter” compared to the “Filter” condition at frontal (*z*(*N* = 13) = −2.06; *p* = 0.039 ^†^; *r* = 0.57) and central (*z*(*N* = 13) = −1.92; *p* = 0.055 ^†^; *r* = 0.53) electrode positions.

### 2.5. Objective Alertness

**Figure 7:**
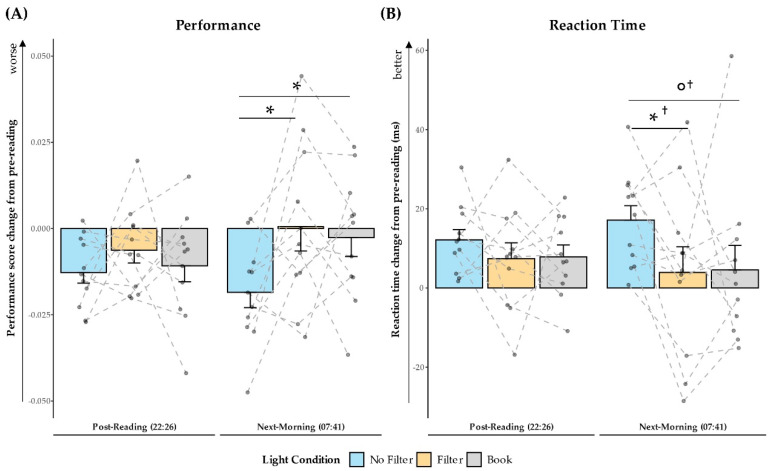
Baseline corrected results for the GO/NOGO task expressed as differences to pre-reading. (**A**): Mean and standard error for the change in median reaction time from pre- to post-reading and from pre-reading to the next morning across conditions with no difference post-reading but slower reaction times during the next morning in the “No Filter” condition. (**B**): Mean and standard error for the change in task performance (see Section 4.8 for the formula) analogously to (**A**) with reduced performance in the “No Filter” condition during the next morning. ° = *p* ≤ 0.10; * = *p* ≤ 0.05; ^†^ = *p*_adj._ > 0.10.

Results from the auditory GO/NOGO task are expressed as change values in reaction time or performance scores from pre-reading to post-reading and to the next morning. No effect of light condition was present in the evening for baseline corrected reaction times (*F*(2,20) = 0.62; *p* = 0.547; *η*^2^ = 0.06) and performance scores (*F*(2,20) = 0.64; *p* = 0.536; *η*^2^ = 0.06). However, the baseline corrected performance scores differed across light conditions in the next morning (*F*(2,20) = 4.62; *p* = 0.022; *η*^2^ = 0.32). Post-hoc analyses indicated that next-morning task performance declined in the “No Filter” condition compared to the “Filter” (*t*(10) = −2.54; *p_adj._* = 0.087; *d* = −0.77) and “Book” (*t*(10) = −2.81; *p_adj._* = 0.057; *d* = −0.85) conditions (cf., Figure 7A). The main effect for light condition on baseline corrected reaction time did not yield significance in the next morning (*F*(2,20) = 2.31; *p* = 0.126; *η*^2^ = 0.19). Nevertheless, explorative post-hoc analyses showed that reaction times were increased in the “No Filter” condition compared to the “Filter” (*t*(10) = 2.29; *p* = 0.045 ^†^; *d* = 0.69) and “Book” (*t*(10) = 1.98; *p* = 0.076 ^†^; *d* = 0.60) condition in the next morning (cf., Figure 7B). For raw data see Supplementary Figure S8.

## 3. Discussion

We assessed the effects of reading on a smartphone with high or reduced amounts of short-wavelength light (without or with a blue light filter) in comparison to reading printed material during the evening. Subjective sleepiness was reduced in the evening during reading and in the next morning in both smartphone conditions compared to reading printed material (cf., Figure 1). However, objective alertness levels were not affected in the evening and only showed an effect in the next morning: contrary to the subjective sleepiness, next-morning alertness was reduced when no filter was used on the smartphone compared to when a blue light filter was switched on or when printed material was used for reading (cf., Figure 7). Salivary cortisol concentration measured immediately after awakening was by trend higher after reading without a blue light filter compared to reading with a filter. However, the CAR (i.e., cortisol increase within the first 30 min post-awakening) was stronger after reading on a smartphone with a filter as compared to reading on a smartphone without a filter (cf., Figure 2), indicating a higher nightly cortisol secretion [22] after reading on a smartphone without a filter. Regarding salivary melatonin levels, concentration was higher during reading on a smartphone without a filter than in both other conditions during reading. Further, it was higher after reading on printed material compared to reading on a smartphone with a filter before sleep (cf., Figure 3). These effects are pointing towards a general light-induced melatonin suppression. In line with these results, the DPG was higher after reading on printed material in comparison to reading on a smartphone with or without a filter around the middle of the night (i.e., at 03:00 and 03:35; cf., Figure 4). These findings indicate less vasodilation of the vessels at distal locations, thus lower heat loss after evening smartphone usage. Furthermore, the different light conditions did not affect whole night sleep architecture but reading on the smartphone without a filter reduced the percentage of SWS and the amount of SWA (0.75–4.5 Hz) during the first night-quarter (cf., Figure 5 and Figure 6). Overall, the negative effects of short-wavelength light on early SWS and next-morning alertness were diminished when a blue light filter was used for reading during the evening. However, the other parameters did not support such a consistent beneficial effect of the blue light filtering software.

### 3.1. Effects on Evening Sleepiness

One possible explanation for the mismatch between the significant effects on subjective sleepiness and the null-effects on objective alertness in the evening could be the difference in assessment times for these parameters. While objective alertness was assessed only approximately 30 min after the light exposure, subjective sleepiness was measured more frequently also during the light exposure, where the effect of the short-wavelength light actually emerged (cf., Figure 1). Thus, the alerting effect of the short-wavelength light while reading on the smartphone without any filter might have only been measurable during the light exposure and could have faded away quickly afterwards, making it undetectable in the subsequent GO/NOGO task (cf., Figure 7). Previous studies also showed the strongest effect of evening short-wavelength light on sleepiness and alertness levels during the light exposure itself [5,14,49]. In one study it was further demonstrated that the distance to the smartphone and the viewing angle can alter and modify the effects of the emitted short-wavelength light [50]. As we did not control the viewing distance and angle during the reading sessions, we cannot eliminate the possibility that differences in these parameters across conditions (e.g., because participants may have held the smartphone in one condition closer to the eyes than in the other condition) could have affected our findings. Another potential moderator could be the display brightness. In our study display brightness was kept constantly at maximum, but different display brightness levels might affect the alerting effects in addition to the spectral characteristics in a similar manner as viewing distance or angle [41,47].

### 3.2. Hormonal Effects

Our results showed a higher melatonin concentration after reading on printed material compared to reading on the smartphone with a blue light filter immediately before sleep (i.e., 50 min after the end of the light exposure; cf., Figure 3). Differences between the smartphone condition without a blue light filter and both other conditions emerged already during reading but pointed into the opposite direction as expected (i.e., higher melatonin levels in the smartphone condition without a blue light filter compared to both other conditions). In order to assure that these results were not only due to a priori differences in melatonin concentrations, data were baseline corrected to pre-light (20:36) melatonin levels (i.e., change from pre-reading melatonin concentration) according to the procedure by Zeitzer and colleagues [51]. After baseline correction, differences during reading on printed material and reading on a smartphone without filter disappeared, whereas differences between the two smartphone conditions during reading were diminished and vanished 30 min later. Furthermore, the baseline corrected data indicated a higher melatonin level after reading on printed material compared to reading on the smartphone with a blue light filter just before bedtime, indicating a bright light-induced melatonin suppression in line with earlier findings [5]. As melatonin is known to initiate heat loss by distal vasodilation of the vessels [27,52], our findings of a higher DPG, indicating a stronger heat loss, around the middle of the night (i.e., at 03:00 and 03:35) after reading on printed material compared to reading in both smartphone conditions (cf., Figure 5) are well in line with these results. Our findings further corroborate previous results that reported a less pronounced nightly CBT course after 3 h of light exposure (~2500 lux) compared to dim light (6 lux) exposure [35]. Furthermore, the presented DPG data show a parallel increase at bedtime in all three light conditions. These thermoregulatory changes preceded by a melatonin rise are robust findings related to psychological cues of relaxation (e.g., supine body posture) [53,54] not time-bound to the evening hours, as they are also reported preceding an afternoon nap [31,53,55]. Regarding our findings, we did not find an immediate light-induced attenuation of thermoregulatory changes (e.g., lower or later DPG increase in the smartphone conditions) at bedtime, despite the fact that melatonin concentration was suppressed at this time after reading on a smartphone with a blue light filter.

### 3.3. Effects on Sleep and Next-Morning Alertness

We observed that especially SWS and SWA during the first night quarter (i.e., the first 2 h of the night) were negatively affected by evening exposure to high portions of short-wavelength light (cf., Figure 5 and Figure 6). These results on SWS and SWA as indices of sleep pressure [56,57] are well in line with existing literature [6,37,38]. This effect was present, even though there was no behavioral effect detectable on objective alertness and subjective sleepiness immediately before bedtime. In our study, almost one hour passed between the end of the light-exposure and the actual bedtime and recent reports suggest that especially many adolescents still use their smartphones or other media devices in bed until only a few minutes before trying to sleep [58,59]. Thus, the actual effects of short-wavelength light on early SWS and SWA might be much stronger in real-world settings. Regarding the refreshing effects of sleep, this sleep impairment could have even further health consequences. Although we found that subjective sleepiness immediately after waking up was higher when no smartphone was used for reading on the preceding evening (cf., Figure 1), objective alertness levels in the smartphone condition without a blue light filter were reduced approximately 30 min later (cf., Figure 7). This suggests that subjects felt less alert after the initial few minutes of awakening in the morning when they were exposed to high amounts of short-wavelength light during the preceding evening. However, the finding that subjective sleepiness was higher during these first minutes after awakening when printed material was used for reading in the preceding evening was unexpected since previous research showed opposite results [9,11,42]. Nevertheless, our findings for objective alertness 30 min after awakening are in line with these previous studies investigating subjective sleepiness.

Furthermore, cortisol concentration at awakening was slightly reduced after reading on the smartphone with a blue light filter compared to reading without a filter. This further resulted in a less pronounced cortisol increase within 30 min post-awakening when no filter was used compared to when the filter was switched on (cf., Figure 3). These results suggest that short-wavelength light in the evening induced heightened SCN-driven cortisol secretion during the night. This assumption is further corroborated by the finding that lower cortisol concentration during the night relates to a stronger CAR [22], whereas a higher CAR in turn relates to lower cortisol levels directly after awakening [23]. Furthermore, it has been concluded in a systematic review that a lower post-awakening increase in cortisol was associated with higher symptoms of fatigue, burnout or exhaustion [60]. Therefore, it would be interesting to conduct further studies investigating the interaction between evening smartphone use and processes of emotion and stress regulation.

In sum, our results indicate that light-induced increases in cortisol concentration throughout the night lead to lower sleepiness levels in the morning directly after awakening, resulting in a lower cortisol increase in response to the transition from sleep to wakefulness within 30 min after awakening.

### 3.4. Limitations and Future Directions

First of all, research on direct and indirect effects of short-wavelength light varies greatly in terms of experimental designs. For our study, a smartphone (Samsung Galaxy A50 enterprise edition; Samsung Electronics, Seoul, Korea) was used to emit different amounts of short-wavelength light and its effects were compared to reading on printed material. Other studies used larger screens [5,61,62], adjustable light-bulbs or lamps [6,12,37,63,64,65] as well as tablet computers [9,11,38,46]. Thus, different devices for the light-exposure will naturally come with different light-intensities at eye level and different spectral properties (i.e., different amounts of short-wavelength light). Due to these differences in study methods and materials, it often seems difficult to compare findings across studies. According to a recent review [66], especially the immediate alerting effects of short-wavelength light appear to be inconsistent between studies. This might be largely due to low powered statistical analyses with small sample sizes (often less than 20 subjects) or between-subjects designs. That critique of course holds true for all other parameters and effects reported in such statistically low powered studies and since we only presented preliminary results with data of 14 participants, our study is no exception to this criticism even though it was conducted as a within-subjects design. Another issue, which is getting increasingly more attention, is that there are large interindividual differences in terms of susceptibility to evening light exposure [67], with some subjects already responding to minimal doses of short-wavelength light and others being almost “immune” to evening light exposure. These differences in light susceptibility arise from a variety of individual differences (e.g., sex, age, chronotype or genetics) and could be an additional threat to the robustness of results from low sample sizes since the chance of selection bias is increased and extreme responders or non-responders might be overrepresented by chance. To overcome these statistical problems, we plan to record and analyze further data to increase the sample size of this study. Thereby we will assess the validity and robustness of the presented preliminary results and additionally we will be able to draw further conclusions. In addition to statistical issues, previous findings showed that bright-light exposure in the morning induced phase advances and earlier sleep onset [68,69]. Therefore, studies investigating effects of evening light exposure always bear a risk for a bias due to different durations and intensities of daylight (i.e., light history) or LED-screen exposure between the subjects before they were examined in the laboratory. This can only be completely avoided constant routine studies where all subjects are immersed in the same environment throughout the whole study duration. However, these studies lack comparability to everyday life situations and the only possibility to control at least to some extent for preceding short-wavelength light exposure in future non-constant routine studies would be to require the subjects to wear blue blocking glasses during the day when they are not in a standardized environment. Regarding our findings, it cannot be ruled out that prior light exposure, especially in the morning, attenuated the short-wavelength light effects during the evening in the laboratory. Furthermore, seasonal differences in daily light exposure between summer and winter have been reported [70], showing an overall lower exposure time to short-wavelength light in winter (November–February) as compared to summer (April–August) in a student population from the UK. In line with these results, findings from Swedish office workers indicate a higher exposure to light radiation of short wavelengths in summer compared to winter, autumn and spring. Additionally, melatonin concentration was higher during winter regarding the peak levels and the concentrations in the morning (07:00) [71]. Our sample was recorded entirely during autumn; therefore, no seasonal effects should have been present between subjects. However, it is unclear if our results might have been different if the study would have been conducted during summer. In total, annual variations in daily light exposure, which influence circadian parameters (e.g., melatonin and cortisol), should be considered when comparing results from different studies investigating the impact of light exposure.

One other limitation could be the relatively long timespan between the end of the reading session and the scheduled bedtime (approximately 1 h) as already discussed before. Even though we still found effects on subsequent sleep, a shorter time period between the light exposure and bedtime could have increased some of the effects. However, since the study was embedded in a larger design in which we also wanted to assess several other parameters after the light-exposure (e.g., alertness levels and questionnaire data), this was unavoidable. Further, we exposed all participants to the same content (i.e., the same reading material) during the light exposure in contrast to some previous studies (e.g., [1]). Thus, the pure effect of evening short-wavelength light emitted by LED-screens was examined. However, different activities on the smartphone (e.g., arousing conversations or watching an arousing movie) could have additional, maybe stronger effects on evening alertness levels and subsequent sleep patterns [39,61,65]. Therefore, the effect of activity could account to some extent for the less clear and pronounced effects shown by our results. One additional feature of smartphones is that they emit electromagnetic fields, which might affect the electrical brain activity on their own that could be reflected in subsequent sleep EEG [72,73]. Future research should try to disentangle the different factors that could be contributing to circadian, sleep and alertness effects of short-wavelength light emitted by smartphones. Lastly, we only tested male young adults which might be a constraint in terms of generalizability of our results and it is therefore unclear whether our findings apply to female subjects or to different age groups. However, by excluding female participants we also eliminated some uncertainty in the data that could have resulted from the female menstrual cycle (e.g., influences on the core body temperature; [74]). There is also evidence for potential sex differences in terms of light perception with men being more sensitive to subjective brightness perception [75], which might affect the alerting response to short-wavelength light. Additionally, age dependent differences have been reported not only in the frame of receiving short-wavelength light information on the retina [76,77], but also in terms of general changes in sleep characteristics and circadian rhythmicity [67,78,79], with older people being in general less sensitive to short-wavelength light.

In sum, the preliminary results of the presented study show that short-wavelength light emitted by a smartphone in the evening has an impact on evening subjective and next-morning objective alertness levels as well as on the circadian rhythm (i.e., reduced CAR, lower nightly DPG and lower melatonin levels before sleep). Furthermore, it affected sleep architecture by reducing SWS and SWA in the first night-quarter. In general, our findings indicate that avoiding any artificial light at night by reading only on printed material is the best strategy since using a blue light filter only slightly attenuated the negative light-induced effects.

## 4. Materials and Methods

### 4.1. Participants

Data were collected from 14 healthy male subjects (*M* = 21.93 years; *SD* = 2.17 years; range = 18–25 years) at the University of Salzburg between October and December 2019. All subjects gave written informed consent and were free of medication, non-smokers and reported no history of neurological or psychiatric diseases. Exclusion criteria also included left-handedness, history of drug abuse, night-shift working, above average caffeine consumption (more than three cups per day) and extreme chronotype. Chronotype was assessed with the German version of the Morning-Eveningness Questionnaire [80]. Fulfillment of inclusion criteria was verified before study enrollment with an online questionnaire that was presented online via LimeSurvey (LimeSurvey: An Open-Source survey tool, LimeSurvey GmbH, Hamburg, Germany). Sleep habits were monitored with wrist actigraphy (Cambridge Neurotechnology Actiwatch©, CamNtech Ltd., Cambridge, UK) and a sleep diary (adapted by [81]) to assure for a regular sleep-wake cycle. Participants were remunerated either with 100 Euro and 16 h University course credit or 50 Euro and 24 h University course credit. The study was approved by the local ethics committee (approval code: GZ16/2014) and was conducted in accordance with the Declaration of Helsinki.

### 4.2. Protocol

**Figure 8:**
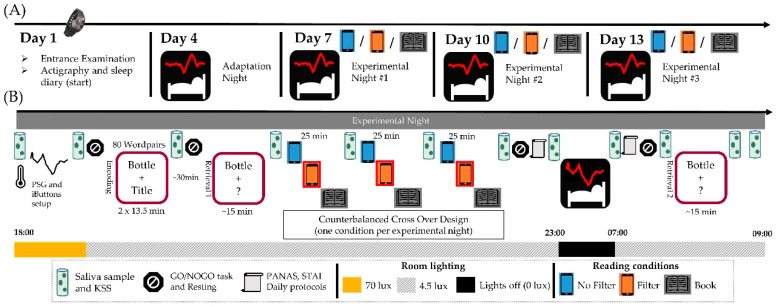
Overview of the study design. (**A**): Outline of the general procedure and the days on which participants had to come to the laboratory. (**B**): Detailed procedure for the experimental nights. Subjects wore wrist actigraphy and completed sleep diaries for the whole study period.

The study design covered a period of 13 days per subject (cf., Figure 8A). On the first day, subjects came to the laboratory for an entrance examination to receive all instructions for their participation. On the fourth day, participants arrived at the laboratory at 21:00 for an adaptation night in order to familiarize themselves with the new sleep environment and the polysomnography recording. A standardized smartphone (Samsung Galaxy A50 enterprise edition; Samsung Electronics, Seoul, Korea) was provided for the participants during all visits. When the subjects had to use the lab-smartphone for completing daily evening and morning questionnaires or when they left the lab, orange-tilted glasses (Uvex Skyper Blue Light Blocking Computer Glasses; Honeywell, Charlotte, NC, USA) were worn. During polysomnography (PSG) montage customized room lights (provided by Emilum GmbH, Oberalm, Austria) were set to 70 photopic lux. Afterwards, room lighting was dimmed to 4.5 photopic lux and a 3 min resting session with eyes closed along with another 3 min with eyes open as well as an auditory GO/NOGO task [82] were carried out. Before and after bedtime, positive (PA) and negative (NA) affectivity (PANAS) [83] as well as state anxiety (STAI-S) [84] were measured. Lights were turned off and participants went to bed around 23:00. The subjects were awakened 8 h after the polysomnography recording started. Approximately 30 min after awakening, resting sessions along with the GO/NOGO task were performed again. Saliva samples were taken, and subjective sleepiness was rated with the KSS [48] regularly throughout the entire visit as indicated in Figure 8B for the experimental recordings.

The experimental recordings took place on day seven, ten and thirteen. Recordings started at 18:00 on each of these days and the procedure of PSG montage and general room light adjustment was the same as for the adaptation night (cf., Figure 8B). First, a resting session together with the GO/NOGO task took place. This was followed by two encoding blocks of a declarative learning task (adapted from Schabus and colleagues [85]). In this version of the task, participants were presented with 80 word-pairs that were displayed once per encoding block. After another resting and GO/NOGO session, a cued retrieval test of the learned word pairs was scheduled. Thereafter, participants read three different stories for a duration of 25 min each. Printed books or the standardized lab-smartphone with either a blue light filter switched off or on were used for reading (cf., Section 4.3). The order of the light conditions was counterbalanced across nights. Afterwards, participants performed another resting and GO/NOGO session. Before going to bed at around 23:00, the PANAS, STAI-S and the daily evening protocol were administered. Participants were awakened exactly 8 h after lights off and the PANAS, STAI-S were administered again along with the daily morning protocol. A final resting and GO/NOGO session were completed. Participants then performed the second cued retrieval from the declarative learning task. Saliva samples were collected together with subjective sleepiness ratings on the KSS at 12 fixed time points throughout each experimental recording. Hence, a total of 42 saliva samples were taken, including six samples from the adaptation night.

### 4.3. Light Exposure (Reading Session)

During each reading session, participants read three different stories in a counterbalanced order. The following stories were used: “Die Känguru Chroniken: Ansichten eines vorlauten Beuteltiers” [86], “555 populäre Irrtümer: Warum Angela Merkel eigentlich ein Wessi ist, man Eier nicht abschrecken muss und Erdnüsse keine Nüsse sind” [87] and “13 gegen das Sommerloch: 13 Autoren–13 Geschichten–13 x Lesespaß” [88]. All stories were presented as e-book versions in the two smartphone conditions “No Filter” and “Filter”. For reading in the condition “Book”, a custom-built ring binder was created with the e-book template to achieve the same layout on paper as on the smartphone. Each story was read for 25 min. Between each story a saliva sample was collected, and the subjects rated their sleepiness (KSS administration). The experimenter verbally asked a few easy and standardized questions about the content of the stories to ensure reading-compliance. Display brightness was always kept at maximum. The display characteristics of the smartphone differed between the “No Filter” and “Filter” condition in terms of the built-in “blue light filter” option which was either switched off or on. The display in the “No Filter” condition had a melanopic radiance of 286.77 mW*m^−2^*sr with a correlated color temperature (CCT) of 8298 K, whereas the display in the “Filter” condition had a melanopic radiance of 114.20 mW*m^−2^*sr and a CCT of 3032 K (for a detailed report generated by the web application “luox.app”, which is meeting the recommended reporting guidelines [89], see Supplementary Report S1). These light measurements were conducted at eye level with a spectrometer (JETI spectraval 1501; JETI Technische Instrumente GmbH, Jena, Germany) that was aimed at the reading stimulus (smartphone or book) while sitting in a chair (height from floor to eye-level: 87 cm, distance from eyes to stimulus: 37 cm). During the light measurements and the actual reading sessions, the dim background room lighting had a melanopic irradiance of 0.42 mW*m^−2^ and a CCT of 2187 K (a detailed report along with measurements of the two smartphone conditions including background room lighting can be found in Supplementary Report S2). Room and smartphone light characteristics were also rated by the subjects (cf., Supplementary Table S1).

### 4.4. Polysomnography

Gold cup electrodes (Grass Technologies, Astro-Med GmbH, Rodgau, Germany) were applied on the following scalp positions: F3, Fz, F4, C3, Cz, C4, P3, Pz, P4, O1 and O2. Fpz served as the ground electrode. Data were offline re-referenced to A1 and A2 (mastoids). Additionally, four electrooculography electrodes (vertical and horizontal) and two electromyography electrodes were placed for PSG. Five temperature buttons (iButton DS1922L; Maxim Integrated Products, Inc., San Jose, CA, USA) were placed on the following positions: right and left infraclavicular, right and left ankle and below the headbox of the EEG system (room) to measure the ambient temperature.

Data was recorded and digitized with a 64 channel BrainAmp system (AMP0305164 Standard; Brain Products GmbH, Munich, Germany), using a sampling rate of 500 Hz. Processing of the data was performed with the BrainVision Recorder (Version 2.11, Brain Products GmbH, 2015) software. Impedances were kept below 10 kΩ throughout all recordings. Sleep stages were automatically classified (Somnolyzer 24 × 7, Version 1.8.1, The Siesta Group, Vienna, Austria) and visually cross-validated by an expert based on the criteria proposed by the American Academy of Sleep Medicine (AASM) [90]. Results of the sleep staging were used for an overview on the general sleep architecture across nights and for further analyses on the time spent in SWS.

Power analyses during the night were conducted with BrainVision Analyzer (Version 2.1.2, Brain Products GmbH, 2017). Data was first pre-processed by implementing a 70 Hz low-pass filter, a 50 Hz notch filter, and a 0.1 Hz high-pass filter. Infinite impulse response (IIR) zero phase shift Butterworth filters were used. Thereafter, an automatic artifact detection was implemented. Only N2 and N3 sleep data was selected for the slow wave activity analysis and segments containing artefacts were removed. Power spectra were computed with Welch’s Method of the Fast Fourier Transform (FFT) by using a sliding hamming window function. Epoch lengths for the averaged power spectra over a total time window were chosen based on the lowest frequency of interest (as recommended by [91]). This resulted in a 2700 ms epoch length for the SWA range (0.75–4.5 Hz).

Results were averaged for further analyses over frontal (F3, Fz, F4), central (C3, Cz, C4), parietal (P3, Pz, P4) and occipital (O1, O2) positions. Average frequency range power was defined as the average amount of power within a given frequency range (i.e., 0.75–4.5 Hz for SWA). Night quarters were used for analyses of temporal dynamics during sleep since the amount and duration of sleep cycles varied across individuals and the separation into night-quarters yielded was more comparable. Time in SWS and SWA (0.75–4.5 Hz) were reported in detail only for the first night-quarter (i.e., the first 2 h of the night) since effects of pre bedtime short-wavelength light were shown to be most pronounced in early sleep [6,37].

### 4.5. Subjective Sleepiness

Situational self-reported sleepiness was assessed verbally with the KSS. The scale consists of one item (“How sleepy do you feel at the moment?”) with a nine-point Likert rating scale (1 = extremely alert; 5 = neither alert nor sleepy; 9 = extremely sleepy, cannot stay awake).

### 4.6. Temperature

Skin temperature was measured continuously in 5 min intervals using external skin sensors (iButton DS1922L; Maxim Integrated Products, Inc., San Jose, CA, USA) with two being placed in an infraclavicular position on the right and left body side (i.e., proximal location relative to the body center), and two on the left and right ankle (i.e., distal location relative to the body center). For further analyses, skin temperature was averaged for each sampling point across proximal and distal positions starting at 19:55. After this, the DPG, which is an indirect measure for heat loss, subsequently leading to a decrease of CBT [92], was computed by subtracting the averaged proximal temperature values from the averaged distal temperature values at each sampling point. After this, the grand average of all DPG values was subtracted from every recorded DPG value in a pointwise fashion as suggested in the literature [93]. Subsequently, we searched for the DPG values that were closest to the KSS applications and saliva samplings and averaged the respective DPG values with the three values preceding and following this DPG values (i.e., average of seven DPG values per time point spanning a time interval of 35 min). During the night, the participants’ DPG values were also averaged across 35 min (c.f., Figure 4 depicted time points ± 15 min).

### 4.7. Melatonin and Cortisol

All saliva samples were stored at −20.0 °C and centrifuged twice to remove mucus and solid particles, first for 15 min followed by another 10 min, before they were refrozen again at −20.0 °C. Salivary melatonin was analyzed using SALIMETRICS salivary melatonin enzyme immunoassay kits (Salimetrics Europe, Suffolk, UK). Salivary cortisol was assessed using the DeMediTec cortisol free in saliva ELISA (Demeditec Diagnostics GmbH, Kiel, Germany). Non-detectable concentrations, i.e., concentrations that were lower than the lowest standard, were set to 0. Missing values (e.g., due to missing samples) were replaced by the mean of the previous and the following concentration, except for missing values right before sleep and after awakening. For these cases missing values were replaced by the sample mean. CAR was defined by the cortisol increase from awakening to 30 min after awakening by calculating the difference of the absolute values between these two time points.

### 4.8. Alertness

An auditory GO/NOGO task was used to assess objective alertness levels. To rule out a priori differences, statistical analyses on this task were conducted on baseline corrected reaction times and performance scores. The task measures inhibition control as well as sustained attention by presenting a GO and a NOGO signal. Participants are asked to react as quickly as possible to the GO-signal, while ignoring the NOGO signal [82]. The task was adapted from Borchard and colleagues [94] and has been implemented with a customized script using the Psychophysics Toolbox Version 3 (PTB-3) in MATLAB (Version R2019b; The MathWorks Inc., Natick, USA). In our version, each assessment comprised 400 trials.

Each trial consisted of a tone (GO or NOGO signal) presented for 50 ms, followed by a randomly varying interstimulus interval between 1480 ms and 1880 ms. The presented tone was either low (1000 Hz) or high (1500 Hz) pitched. In 80% of the trials, the GO-tone (1000 or 1500 Hz) was presented. During the beginning of each run, it was determined by a 50% chance whether the high- or low-pitched tone served as the GO-signal for the subsequent assessment. Participants were asked to react with their left thumb on one of the buttons of a response time box (RTBox v5/6; distributed by the Ohio State University, Columbus, OH, USA). Median reaction times of all valid trials (i.e., excluding commission errors) and overall task performance were assessed. Task performance was calculated by the following formula (adapted from Figueiro and colleagues [63]) with higher values indicating better performance:

(1) $$P\left(k\right)=\;\frac{100*\left(\frac{V}{T}\right)}{R}$$

The variables refer to: *k* = 1, 2, …, *n* corresponding to the performance score in a single GO/NOGO task session. *V* represents the number of valid responses which were not allowed to be commission errors, lapses or false alarms (reactions faster than 150 ms). *T* refers to the amount of total trials and *R* corresponds to the median of all valid reaction times in ms throughout the session *k.*

### 4.9. Statistical Analyses

For statistical analyses SPSS (Version 26; IBM Corp., Armonk, NY, USA) and R-Studio (Version 3.6.1) were used. Repeated measures analyses of variance (ANOVAs) with follow-up pairwise comparisons were implemented when assumptions were met. Significant violations of the sphericity assumption for repeated measures ANOVAs were accounted for with Greenhouse–Geisser adjustments for *ε* ≤ 0.75 or Huynh–Feldt adjustments for *ε* > 0.75 [95]. An observation was treated as an outlier and was excluded from parametric analyses, if it had a distance of more than three times the interquartile range to the upper or lower quartile. If normality was still violated after exclusion of the outlier(s), non-parametric Friedman tests with follow-up Wilcoxon tests were conducted on the full sample. Even though only a preliminary dataset was presented (*N* = 14), a statistical power of approximately 80% was achievable for within-subjects comparisons on single dependent variables if all measurement points were available.

## Figures and Tables

**Table 1:** Median and interquartile ranges (IQR) for whole night sleep architecture. Chi-Square test statistics and significance levels are derived from non-parametric Friedman tests (*N* = 13).

	No Filter	Filter	Book	χ^2^	*p*
TIB (min)	480.50 (0.50)	480.50 (1.00)	480.50 (1.00)	1.72	0.423
TST (min)	466.00 (34.00)	468.00 (48.00)	469.00 (11.75)	2.46	0.292
SEFF (%)	96.98 (7.42)	97.40 (9.98)	97.51 (2.59)	1.92	0.383
SOL N2 (min)	13.50 (6.75)	11.00 (12.25)	11.00 (6.75)	0.28	0.869
N1 (%)	10.13 (7.24)	11.42 (7.51)	10.49 (7.55)	2.46	0.292
N2 (%)	39.66 (4.33)	38.35 (8.85)	40.44 (10.04)	3.23	0.199
N3 (%)	28.53 (9.88)	31.65 (10.73)	25.45 (9.50)	1.92	0.383
REM (%)	19.25 (6.25)	20.18 (4.78)	20.90 (6.66)	0.46	0.794
WASO (min)	12.00 (26.25)	6.50 (41.50)	7.00 (9.25)	1.92	0.383

Note. TIB = Time in bed, TST = Total sleep time, SEFF = Sleep efficiency, SOL N2 = Sleep onset latency to N2, WASO = Wake after sleep onset.

## Supplementary Materials

The following are available online at https://www.mdpi.com/2624-5175/3/1/5/s1. Figure S1: Cortisol increase differences, Figure S2: Melatonin baseline corrected results, Figure S3: Time in SWS for whole night, Figure S4: SWA for second night-quarter, Figure S5: SWA for third night-quarter, Figure S6: SWA for last night-quarter, Figure S7: SWA for whole night, Figure S8: GO/NOGO raw results, Report S1: Smartphone light measurements, Report S2: Room light measurements, Table S1: subjective light rating.

## Abbreviations

ANOVA	Analysis of variance
CAR	Cortisol awakening response
CBT	Core body temperature
DPG	Distal proximal gradient
ipRGC	Intrinsically photosensitive retinal ganglion cell
IQR	Interquartile range
KSS	Karolinska Sleepiness Scale
PSG	Polysomnography
SCN	Suprachiasmatic nucleus
SWA	Slow wave activity
SWS	Slow wave sleep
