# Supplementary material for: Preliminary Results: The Impact of Smartphone Use and Short-Wavelength Light during the Evening on Circadian Rhythm, Sleep and Alertness
Source: Clocks Sleep. 2021 Jan 22;3(1):66–86. doi: 10.3390/clockssleep3010005 (PMC7838958; doi:10.3390/clockssleep3010005)
Supplement: Supplementary file 1 [file clockssleep-03-00005-s001.zip › Report_S1.pdf]

Reporting Light Exposure

Step 1. Upload your spectral power distribution data.

Choose file... Browse

Step 2. Tell us more about your data.

My data contains [absolute] spectra with wavelength in nm. My measurements are labelled [No Filter] and [Filter]. Each measurement column contains [radiances] in [W] per [m²] per sr.

Step 3. Check we have understood your input correctly.

Check that we have loaded the correct number of observations. Also check the units of measurement. It may help to compare your spectra to a standard reference spectra. For example, if your measurements were made in daylight, check that they have a similar shape to `CIE Standard Illuminant D65`. Your data might be easier to read if you change the y-axis scale.

Y-axis scale

- (•) Raw data
- ( ) Normalised data
- ( ) Log10

Reference spectrum

Reference spectra

[Select a reference spectrum]

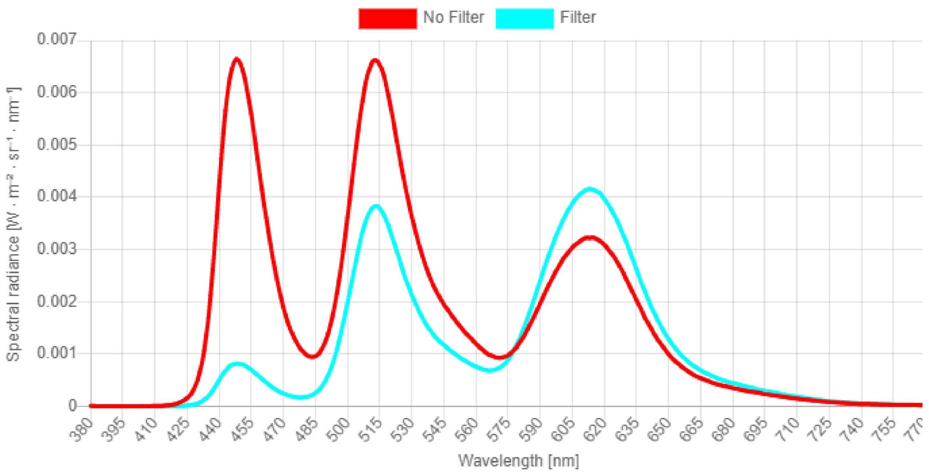

Step 4. Download the stimulus specification tables and include them in your report.

☐ Use exponential notation? ☐ Show advanced calculations? [Download CSV]

Condition	No Filter	Filter
**Luminance [cd/m²]**	183.9575	142.3532
**Colour rendering index (CIE Ra)**	82.5000	73.3750
**S-cone-opic radiance (mW · m⁻² · sr)**	168.1941	24.9606
**M-cone-opic radiance (mW · m⁻² · sr)**	284.3734	175.3251
**L-cone-opic radiance (mW · m⁻² · sr)**	298.4939	236.8602
**Rhodopic radiance (mW · m⁻² · sr)**	317.1473	144.3093
**Melanopic radiance (mW · m⁻² · sr)**	286.7672	114.2011

Step 5. Include the full spectral power distribution in your supplementary material.

Where a journal does not offer the capability of making Supplementary Material available, files can be made available on [Figshare], the [Open Science Framework] or [GitHub]. Some institutions also offer repositories for research data.

☑ Use exponential notation? [Download CSV]

Wavelength [nm]		Spectral radiance [$W \cdot m^{-2} \cdot sr^{-1} \cdot nm^{-1}$]
**380**	1.25e-05	2.30e-06
**381**	1.03e-05	0.00e+00
**382**	6.67e-06	0.00e+00
**383**	3.05e-06	0.00e+00
**384**	1.70e-06	4.87e-08
**385**	4.68e-06	2.14e-07
**386**	4.98e-06	1.71e-07
**387**	3.70e-06	6.86e-09
**388**	4.69e-06	0.00e+00
**389**	5.26e-06	0.00e+00
**390**	5.02e-06	0.00e+00
**391**	4.70e-06	0.00e+00
**392**	4.32e-06	0.00e+00
**393**	2.67e-06	0.00e+00
**394**	1.93e-06	2.82e-08
**395**	3.40e-06	1.21e-07
**396**	3.90e-06	9.20e-08
**397**	3.88e-06	3.49e-09
**398**	2.97e-06	7.35e-08
**399**	2.45e-06	2.70e-07
**400**	2.68e-06	7.52e-07
**401**	3.59e-06	7.43e-07
**402**	4.87e-06	4.62e-07
**403**	5.41e-06	1.55e-06
**404**	5.77e-06	2.39e-06
**405**	5.80e-06	2.64e-06
**406**	5.31e-06	2.09e-06
**407**	4.57e-06	1.13e-06
**408**	5.99e-06	1.78e-06
**409**	7.24e-06	1.99e-06
**410**	8.12e-06	1.26e-06
**411**	8.63e-06	1.02e-06
**412**	8.98e-06	1.01e-06
**413**	1.07e-05	7.63e-07
**414**	1.34e-05	1.19e-06
**415**	1.80e-05	2.81e-06
**416**	2.12e-05	3.68e-06
**417**	2.40e-05	4.24e-06
**418**	3.18e-05	4.65e-06
**419**	4.03e-05	5.34e-06
**420**	4.96e-05	6.45e-06
**421**	6.52e-05	7.06e-06
**422**	8.34e-05	7.75e-06
**423**	1.04e-04	1.11e-05
**424**	1.28e-04	1.45e-05
**425**	1.59e-04	1.77e-05
**426**	1.98e-04	2.21e-05

Wavelength [nm]		Spectral radiance [W · m^{-2} · sr^{-1} · nm^{-1}]
**427**	2.44e-04	2.74e-05
**428**	3.16e-04	3.61e-05
**429**	4.05e-04	4.58e-05
**430**	5.12e-04	5.66e-05
**431**	6.61e-04	7.56e-05
**432**	8.32e-04	9.76e-05
**433**	1.07e-03	1.26e-04
**434**	1.36e-03	1.58e-04
**435**	1.69e-03	1.95e-04
**436**	2.13e-03	2.45e-04
**437**	2.59e-03	3.01e-04
**438**	3.11e-03	3.64e-04
**439**	3.65e-03	4.32e-04
**440**	4.20e-03	5.03e-04
**441**	4.73e-03	5.72e-04
**442**	5.23e-03	6.34e-04
**443**	5.66e-03	6.81e-04
**444**	6.00e-03	7.27e-04
**445**	6.28e-03	7.73e-04
**446**	6.45e-03	7.94e-04
**447**	6.58e-03	8.09e-04
**448**	6.64e-03	8.16e-04
**449**	6.62e-03	8.15e-04
**450**	6.55e-03	8.11e-04
**451**	6.43e-03	8.01e-04
**452**	6.28e-03	7.84e-04
**453**	6.06e-03	7.56e-04
**454**	5.83e-03	7.29e-04
**455**	5.60e-03	7.03e-04
**456**	5.31e-03	6.67e-04
**457**	5.02e-03	6.33e-04
**458**	4.73e-03	5.98e-04
**459**	4.43e-03	5.64e-04
**460**	4.15e-03	5.30e-04
**461**	3.87e-03	4.93e-04
**462**	3.61e-03	4.59e-04
**463**	3.35e-03	4.27e-04
**464**	3.10e-03	3.93e-04
**465**	2.86e-03	3.61e-04
**466**	2.64e-03	3.36e-04
**467**	2.44e-03	3.10e-04
**468**	2.24e-03	2.84e-04
**469**	2.07e-03	2.66e-04
**470**	1.90e-03	2.49e-04
**471**	1.75e-03	2.34e-04
**472**	1.62e-03	2.19e-04
**473**	1.50e-03	2.04e-04

Wavelength [nm]		Spectral radiance [W · m^{-2} · sr^{-1} · nm^{-1}]
**474**	1.41e-03	1.94e-04
**475**	1.32e-03	1.85e-04
**476**	1.24e-03	1.77e-04
**477**	1.17e-03	1.73e-04
**478**	1.10e-03	1.72e-04
**479**	1.06e-03	1.73e-04
**480**	1.02e-03	1.77e-04
**481**	9.80e-04	1.84e-04
**482**	9.60e-04	1.96e-04
**483**	9.48e-04	2.10e-04
**484**	9.49e-04	2.32e-04
**485**	9.61e-04	2.59e-04
**486**	9.84e-04	2.91e-04
**487**	1.03e-03	3.33e-04
**488**	1.10e-03	3.81e-04
**489**	1.18e-03	4.45e-04
**490**	1.28e-03	5.18e-04
**491**	1.40e-03	5.99e-04
**492**	1.56e-03	7.00e-04
**493**	1.73e-03	8.10e-04
**494**	1.93e-03	9.38e-04
**495**	2.16e-03	1.08e-03
**496**	2.40e-03	1.24e-03
**497**	2.68e-03	1.42e-03
**498**	2.98e-03	1.60e-03
**499**	3.29e-03	1.78e-03
**500**	3.62e-03	1.98e-03
**501**	3.95e-03	2.19e-03
**502**	4.29e-03	2.39e-03
**503**	4.63e-03	2.59e-03
**504**	4.97e-03	2.79e-03
**505**	5.28e-03	2.98e-03
**506**	5.58e-03	3.17e-03
**507**	5.84e-03	3.34e-03
**508**	6.08e-03	3.48e-03
**509**	6.29e-03	3.60e-03
**510**	6.42e-03	3.69e-03
**511**	6.53e-03	3.76e-03
**512**	6.61e-03	3.81e-03
**513**	6.62e-03	3.83e-03
**514**	6.60e-03	3.83e-03
**515**	6.53e-03	3.79e-03
**516**	6.45e-03	3.73e-03
**517**	6.33e-03	3.66e-03
**518**	6.15e-03	3.57e-03
**519**	5.96e-03	3.47e-03
**520**	5.76e-03	3.35e-03

Wavelength [nm]		Spectral radiance [W $\cdot$ m^{-2} $\cdot$ sr^{-1} $\cdot$ nm^{-1}]
**521**	5.55e-03	3.22e-03
**522**	5.31e-03	3.10e-03
**523**	5.08e-03	2.97e-03
**524**	4.85e-03	2.85e-03
**525**	4.62e-03	2.71e-03
**526**	4.41e-03	2.58e-03
**527**	4.20e-03	2.45e-03
**528**	4.01e-03	2.35e-03
**529**	3.82e-03	2.25e-03
**530**	3.63e-03	2.13e-03
**531**	3.47e-03	2.04e-03
**532**	3.31e-03	1.95e-03
**533**	3.15e-03	1.86e-03
**534**	3.01e-03	1.77e-03
**535**	2.88e-03	1.70e-03
**536**	2.75e-03	1.62e-03
**537**	2.63e-03	1.55e-03
**538**	2.52e-03	1.49e-03
**539**	2.42e-03	1.44e-03
**540**	2.32e-03	1.38e-03
**541**	2.24e-03	1.33e-03
**542**	2.16e-03	1.29e-03
**543**	2.09e-03	1.24e-03
**544**	2.02e-03	1.21e-03
**545**	1.95e-03	1.18e-03
**546**	1.89e-03	1.14e-03
**547**	1.84e-03	1.10e-03
**548**	1.78e-03	1.07e-03
**549**	1.72e-03	1.04e-03
**550**	1.67e-03	1.01e-03
**551**	1.61e-03	9.82e-04
**552**	1.56e-03	9.50e-04
**553**	1.51e-03	9.17e-04
**554**	1.46e-03	8.95e-04
**555**	1.41e-03	8.72e-04
**556**	1.37e-03	8.49e-04
**557**	1.33e-03	8.24e-04
**558**	1.29e-03	7.99e-04
**559**	1.24e-03	7.83e-04
**560**	1.20e-03	7.64e-04
**561**	1.16e-03	7.43e-04
**562**	1.12e-03	7.27e-04
**563**	1.09e-03	7.12e-04
**564**	1.05e-03	7.03e-04
**565**	1.02e-03	6.94e-04
**566**	9.90e-04	6.85e-04
**567**	9.77e-04	6.88e-04

Wavelength [nm]		Spectral radiance [W $\cdot$ m^{-2} $\cdot$ sr^{-1} $\cdot$ nm^{-1}]
**568**	9.61e-04	6.94e-04
**569**	9.42e-04	7.05e-04
**570**	9.33e-04	7.20e-04
**571**	9.29e-04	7.37e-04
**572**	9.35e-04	7.67e-04
**573**	9.45e-04	8.01e-04
**574**	9.57e-04	8.40e-04
**575**	9.79e-04	8.94e-04
**576**	1.01e-03	9.53e-04
**577**	1.05e-03	1.02e-03
**578**	1.09e-03	1.10e-03
**579**	1.13e-03	1.18e-03
**580**	1.20e-03	1.26e-03
**581**	1.26e-03	1.36e-03
**582**	1.32e-03	1.46e-03
**583**	1.39e-03	1.57e-03
**584**	1.47e-03	1.68e-03
**585**	1.55e-03	1.79e-03
**586**	1.63e-03	1.91e-03
**587**	1.71e-03	2.03e-03
**588**	1.80e-03	2.15e-03
**589**	1.88e-03	2.28e-03
**590**	1.97e-03	2.40e-03
**591**	2.06e-03	2.53e-03
**592**	2.15e-03	2.66e-03
**593**	2.24e-03	2.78e-03
**594**	2.32e-03	2.89e-03
**595**	2.40e-03	3.00e-03
**596**	2.48e-03	3.11e-03
**597**	2.56e-03	3.22e-03
**598**	2.63e-03	3.32e-03
**599**	2.70e-03	3.41e-03
**600**	2.77e-03	3.50e-03
**601**	2.84e-03	3.59e-03
**602**	2.89e-03	3.67e-03
**603**	2.94e-03	3.75e-03
**604**	2.99e-03	3.81e-03
**605**	3.04e-03	3.88e-03
**606**	3.08e-03	3.94e-03
**607**	3.11e-03	3.99e-03
**608**	3.14e-03	4.03e-03
**609**	3.17e-03	4.07e-03
**610**	3.20e-03	4.10e-03
**611**	3.22e-03	4.13e-03
**612**	3.23e-03	4.15e-03
**613**	3.22e-03	4.16e-03
**614**	3.23e-03	4.15e-03

Wavelength [nm]		Spectral radiance [$W \cdot m^{-2} \cdot sr^{-1} \cdot nm^{-1}$]
**615**	3.23e-03	4.14e-03
**616**	3.21e-03	4.12e-03
**617**	3.19e-03	4.10e-03
**618**	3.16e-03	4.08e-03
**619**	3.13e-03	4.02e-03
**620**	3.09e-03	3.97e-03
**621**	3.05e-03	3.91e-03
**622**	2.99e-03	3.85e-03
**623**	2.94e-03	3.78e-03
**624**	2.89e-03	3.70e-03
**625**	2.83e-03	3.63e-03
**626**	2.76e-03	3.55e-03
**627**	2.69e-03	3.46e-03
**628**	2.62e-03	3.36e-03
**629**	2.54e-03	3.26e-03
**630**	2.45e-03	3.17e-03
**631**	2.37e-03	3.06e-03
**632**	2.28e-03	2.95e-03
**633**	2.20e-03	2.84e-03
**634**	2.12e-03	2.73e-03
**635**	2.03e-03	2.62e-03
**636**	1.95e-03	2.51e-03
**637**	1.87e-03	2.39e-03
**638**	1.78e-03	2.29e-03
**639**	1.69e-03	2.20e-03
**640**	1.62e-03	2.09e-03
**641**	1.55e-03	1.99e-03
**642**	1.48e-03	1.89e-03
**643**	1.40e-03	1.81e-03
**644**	1.34e-03	1.73e-03
**645**	1.27e-03	1.64e-03
**646**	1.22e-03	1.57e-03
**647**	1.16e-03	1.49e-03
**648**	1.11e-03	1.42e-03
**649**	1.05e-03	1.35e-03
**650**	1.00e-03	1.29e-03
**651**	9.51e-04	1.23e-03
**652**	9.06e-04	1.17e-03
**653**	8.67e-04	1.12e-03
**654**	8.35e-04	1.07e-03
**655**	8.04e-04	1.02e-03
**656**	7.64e-04	9.73e-04
**657**	7.32e-04	9.35e-04
**658**	7.05e-04	9.02e-04
**659**	6.76e-04	8.61e-04
**660**	6.48e-04	8.24e-04
**661**	6.21e-04	7.95e-04

Wavelength [nm]		Spectral radiance [$W \cdot m^{-2} \cdot sr^{-1} \cdot nm^{-1}$]
**662**	5.96e-04	7.68e-04
**663**	5.73e-04	7.42e-04
**664**	5.58e-04	7.17e-04
**665**	5.42e-04	6.92e-04
**666**	5.24e-04	6.64e-04
**667**	5.09e-04	6.45e-04
**668**	4.94e-04	6.27e-04
**669**	4.79e-04	6.09e-04
**670**	4.61e-04	5.89e-04
**671**	4.44e-04	5.70e-04
**672**	4.32e-04	5.53e-04
**673**	4.21e-04	5.36e-04
**674**	4.11e-04	5.21e-04
**675**	4.00e-04	5.05e-04
**676**	3.90e-04	4.91e-04
**677**	3.83e-04	4.80e-04
**678**	3.74e-04	4.68e-04
**679**	3.64e-04	4.56e-04
**680**	3.51e-04	4.47e-04
**681**	3.43e-04	4.38e-04
**682**	3.38e-04	4.29e-04
**683**	3.27e-04	4.17e-04
**684**	3.17e-04	4.05e-04
**685**	3.09e-04	3.90e-04
**686**	3.01e-04	3.82e-04
**687**	2.92e-04	3.75e-04
**688**	2.86e-04	3.66e-04
**689**	2.80e-04	3.56e-04
**690**	2.74e-04	3.46e-04
**691**	2.70e-04	3.32e-04
**692**	2.64e-04	3.21e-04
**693**	2.53e-04	3.16e-04
**694**	2.44e-04	3.07e-04
**695**	2.36e-04	2.97e-04
**696**	2.31e-04	2.89e-04
**697**	2.25e-04	2.82e-04
**698**	2.18e-04	2.75e-04
**699**	2.13e-04	2.69e-04
**700**	2.08e-04	2.62e-04
**701**	2.02e-04	2.54e-04
**702**	1.96e-04	2.43e-04
**703**	1.90e-04	2.33e-04
**704**	1.85e-04	2.28e-04
**705**	1.79e-04	2.20e-04
**706**	1.71e-04	2.11e-04
**707**	1.65e-04	2.06e-04
**708**	1.59e-04	2.01e-04

Wavelength [nm]	Spectral radiance [$W \cdot m^{-2} \cdot sr^{-1} \cdot nm^{-1}$]	
**709**	1.56e-04	1.95e-04
**710**	1.49e-04	1.88e-04
**711**	1.43e-04	1.81e-04
**712**	1.38e-04	1.76e-04
**713**	1.34e-04	1.69e-04
**714**	1.30e-04	1.60e-04
**715**	1.26e-04	1.54e-04
**716**	1.22e-04	1.48e-04
**717**	1.17e-04	1.40e-04
**718**	1.13e-04	1.35e-04
**719**	1.10e-04	1.31e-04
**720**	1.05e-04	1.28e-04
**721**	1.00e-04	1.22e-04
**722**	9.54e-05	1.14e-04
**723**	9.28e-05	1.09e-04
**724**	8.95e-05	1.05e-04
**725**	8.52e-05	1.03e-04
**726**	8.25e-05	1.01e-04
**727**	7.99e-05	9.78e-05
**728**	7.68e-05	9.19e-05
**729**	7.35e-05	8.83e-05
**730**	7.02e-05	8.60e-05
**731**	6.80e-05	8.23e-05
**732**	6.55e-05	7.92e-05
**733**	6.28e-05	7.69e-05
**734**	6.05e-05	7.26e-05
**735**	5.83e-05	6.89e-05
**736**	5.62e-05	6.78e-05
**737**	5.46e-05	6.61e-05
**738**	5.31e-05	6.40e-05
**739**	4.87e-05	5.96e-05
**740**	4.64e-05	5.63e-05
**741**	4.62e-05	5.39e-05
**742**	4.71e-05	5.41e-05
**743**	4.73e-05	5.37e-05
**744**	4.59e-05	5.17e-05
**745**	4.31e-05	5.01e-05
**746**	4.04e-05	4.84e-05
**747**	4.00e-05	4.53e-05
**748**	3.90e-05	4.49e-05
**749**	3.74e-05	4.65e-05
**750**	3.74e-05	4.27e-05
**751**	3.63e-05	3.99e-05
**752**	3.34e-05	3.87e-05
**753**	3.27e-05	3.92e-05
**754**	3.23e-05	3.95e-05
**755**	3.15e-05	3.84e-05

Wavelength [nm]		Spectral radiance [W · m^{-2} · sr^{-1} · nm^{-1}]
**756**	3.07e-05	3.64e-05
**757**	3.01e-05	3.42e-05
**758**	3.04e-05	3.43e-05
**759**	2.97e-05	3.40e-05
**760**	2.82e-05	3.33e-05
**761**	2.77e-05	3.28e-05
**762**	2.76e-05	3.27e-05
**763**	2.79e-05	3.31e-05
**764**	2.74e-05	3.20e-05
**765**	2.69e-05	3.10e-05
**766**	2.68e-05	3.11e-05
**767**	2.58e-05	3.00e-05
**768**	2.43e-05	2.83e-05
**769**	2.29e-05	2.62e-05
**770**	2.30e-05	2.57e-05
**771**	2.44e-05	2.61e-05
**772**	2.46e-05	2.53e-05
**773**	2.38e-05	2.52e-05
**774**	2.18e-05	2.60e-05
**775**	2.18e-05	2.56e-05
**776**	2.20e-05	2.49e-05
**777**	2.20e-05	2.41e-05
**778**	2.13e-05	2.29e-05
**779**	2.02e-05	2.22e-05
**780**	1.84e-05	2.46e-05

Step 6. Share an online version of this report.

https://luox.app/u/spd1,380,1,wr,-28,CkCVB4BRA8BkBnBZBkBqBoBkBgBMBABVBbBbBQBJBMBYBmBsBvBwBrBjBxB9CECICLCYCqDFD[illegible]

Click above to copy the URL to your clipboard
