# Supplementary material for: Preliminary Results: The Impact of Smartphone Use and Short-Wavelength Light during the Evening on Circadian Rhythm, Sleep and Alertness
Source: Clocks Sleep. 2021 Jan 22;3(1):66–86. doi: 10.3390/clockssleep3010005 (PMC7838958; doi:10.3390/clockssleep3010005)
Supplement: Supplementary file 1 [file clockssleep-03-00005-s001.zip › Report_S2.pdf]

Reporting Light Exposure

Step 1. Upload your spectral power distribution data.

Choose file... Browse

Step 2. Tell us more about your data.

My data contains absolute spectra with wavelength in nm. My measurements are labelled Room Lighting, No Filter (+ Background) and Filter (+ Background). Each measurement column contains irradiances in W per m².

Step 3. Check we have understood your input correctly.

Check that we have loaded the correct number of observations. Also check the units of measurement. It may help to compare your spectra to a standard reference spectra. For example, if your measurements were made in daylight, check that they have a similar shape to `CIE Standard Illuminant D65`. Your data might be easier to read if you change the y-axis scale.

Y-axis scale

- Raw data
- Normalised data
- Log10

Reference spectrum

Reference spectra

Select a reference spectrum

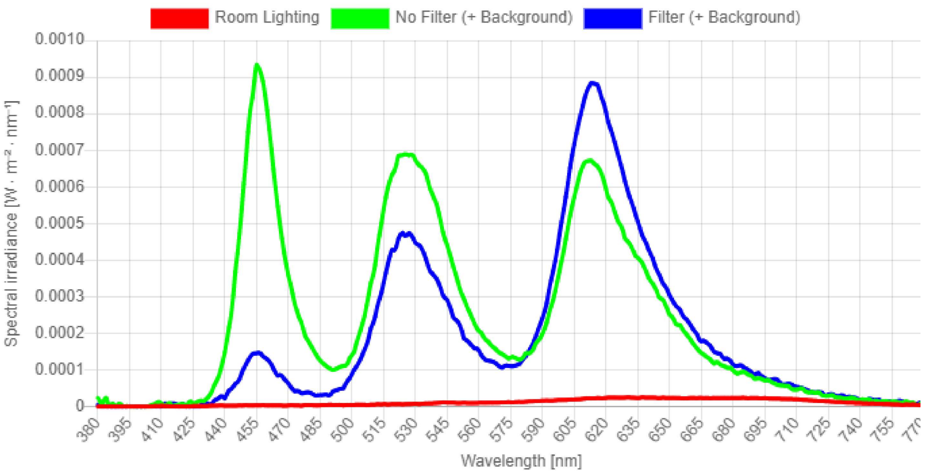

Step 4. Download the stimulus specification tables and include them in your report.

☐ Use exponential notation? ☐ Show advanced calculations? Download CSV

Condition	Room Lighting	No Filter (+ Background)	Filter (+ Background)
**Illuminance [lux]**	0.8811	27.0953	22.7484
**Colour rendering index (CIE Ra)**	95.8750	76.6250	75.7500
**S-cone-opic irradiance (mW · m^{-2})**	0.1466	18.4570	3.3973
**M-cone-opic irradiance (mW · m^{-2})**	0.9278	37.6039	25.5362
**L-cone-opic irradiance (mW · m^{-2})**	1.4901	43.7776	37.8960
**Rhodopic irradiance (mW · m^{-2})**	0.5625	36.3673	17.9821
**Melanopic irradiance (mW · m^{-2})**	0.4162	31.6250	13.1434

Step 5. Include the full spectral power distribution in your supplementary material.

Where a journal does not offer the capability of making Supplementary Material available, files can be made available on Figshare, the Open Science Framework or GitHub. Some institutions also offer repositories for research data.

☑ Use exponential notation?

Download CSV

Wavelength [nm]			Spectral irradiance [W · m^{-2} · nm^{-1}]
**380**	0.00e+00	2.38e-05	4.56e-06
**381**	0.00e+00	1.57e-05	3.02e-06
**382**	0.00e+00	4.20e-07	8.07e-08
**383**	0.00e+00	1.76e-05	2.10e-07
**384**	0.00e+00	2.35e-05	2.79e-07
**385**	0.00e+00	6.48e-06	7.71e-08
**386**	0.00e+00	3.22e-06	0.00e+00
**387**	0.00e+00	8.23e-06	0.00e+00
**388**	0.00e+00	3.18e-06	0.00e+00
**389**	0.00e+00	1.89e-06	0.00e+00
**390**	0.00e+00	7.45e-06	0.00e+00
**391**	0.00e+00	5.61e-06	0.00e+00
**392**	0.00e+00	2.31e-07	0.00e+00
**393**	0.00e+00	7.01e-07	0.00e+00
**394**	0.00e+00	9.89e-07	2.07e-07
**395**	0.00e+00	2.60e-07	8.89e-07
**396**	0.00e+00	0.00e+00	6.73e-07
**397**	0.00e+00	0.00e+00	2.53e-08
**398**	8.87e-08	0.00e+00	0.00e+00
**399**	1.20e-07	0.00e+00	0.00e+00
**400**	3.10e-08	0.00e+00	0.00e+00
**401**	0.00e+00	0.00e+00	5.15e-08
**402**	0.00e+00	0.00e+00	1.31e-07
**403**	0.00e+00	0.00e+00	5.13e-07
**404**	0.00e+00	7.75e-07	6.76e-07
**405**	0.00e+00	3.22e-06	3.56e-07
**406**	6.13e-07	5.24e-06	2.43e-07
**407**	1.54e-06	7.01e-06	2.39e-07
**408**	1.12e-06	1.11e-05	9.65e-08
**409**	1.09e-06	1.26e-05	2.35e-08
**410**	1.88e-06	8.66e-06	9.51e-08
**411**	1.75e-06	4.36e-06	3.61e-06
**412**	1.17e-06	1.06e-07	8.63e-06
**413**	1.02e-06	7.33e-06	4.75e-06
**414**	8.46e-07	9.29e-06	3.95e-06
**415**	6.35e-07	2.11e-06	8.51e-06
**416**	3.77e-07	1.70e-06	8.52e-06
**417**	1.11e-07	4.03e-06	6.62e-06
**418**	4.20e-08	4.90e-06	4.59e-06
**419**	0.00e+00	5.88e-06	4.46e-06
**420**	0.00e+00	7.03e-06	7.29e-06
**421**	0.00e+00	4.94e-06	4.43e-06
**422**	2.51e-08	2.75e-06	0.00e+00
**423**	3.07e-07	1.14e-05	0.00e+00
**424**	6.82e-07	1.39e-05	0.00e+00

Wavelength [nm]			Spectral irradiance [W · m⁻² · nm⁻¹]
**425**	1.18e-06	8.61e-06	0.00e+00
**426**	1.40e-06	9.66e-06	0.00e+00
**427**	1.55e-06	1.25e-05	0.00e+00
**428**	1.69e-06	1.55e-05	0.00e+00
**429**	1.57e-06	1.64e-05	4.63e-06
**430**	1.14e-06	1.51e-05	1.42e-05
**431**	1.35e-06	1.93e-05	1.21e-05
**432**	1.72e-06	2.49e-05	7.98e-06
**433**	2.19e-06	3.21e-05	5.66e-06
**434**	2.48e-06	4.32e-05	8.65e-06
**435**	2.60e-06	5.76e-05	1.62e-05
**436**	2.67e-06	6.86e-05	1.94e-05
**437**	2.78e-06	8.12e-05	2.26e-05
**438**	3.05e-06	1.00e-04	2.78e-05
**439**	2.88e-06	1.23e-04	2.74e-05
**440**	2.39e-06	1.48e-04	2.29e-05
**441**	2.45e-06	1.82e-04	3.41e-05
**442**	2.56e-06	2.16e-04	4.41e-05
**443**	2.71e-06	2.50e-04	4.81e-05
**444**	2.64e-06	3.04e-04	5.45e-05
**445**	2.46e-06	3.67e-04	6.22e-05
**446**	2.81e-06	4.18e-04	6.71e-05
**447**	2.98e-06	4.73e-04	7.55e-05
**448**	2.77e-06	5.39e-04	9.14e-05
**449**	3.14e-06	6.08e-04	1.04e-04
**450**	3.77e-06	6.76e-04	1.15e-04
**451**	3.27e-06	7.49e-04	1.26e-04
**452**	2.98e-06	8.08e-04	1.36e-04
**453**	3.00e-06	8.43e-04	1.43e-04
**454**	3.32e-06	8.88e-04	1.45e-04
**455**	3.68e-06	9.32e-04	1.46e-04
**456**	3.34e-06	9.25e-04	1.47e-04
**457**	3.36e-06	9.11e-04	1.45e-04
**458**	3.83e-06	8.89e-04	1.38e-04
**459**	3.43e-06	8.51e-04	1.33e-04
**460**	2.93e-06	8.08e-04	1.29e-04
**461**	3.17e-06	7.56e-04	1.23e-04
**462**	3.26e-06	7.03e-04	1.15e-04
**463**	3.20e-06	6.48e-04	1.03e-04
**464**	3.18e-06	5.96e-04	9.27e-05
**465**	3.17e-06	5.46e-04	8.48e-05
**466**	3.10e-06	5.01e-04	8.67e-05
**467**	2.78e-06	4.60e-04	8.34e-05
**468**	2.24e-06	4.20e-04	7.58e-05
**469**	2.53e-06	3.88e-04	6.97e-05
**470**	2.99e-06	3.58e-04	6.31e-05
**471**	3.70e-06	3.35e-04	5.48e-05

,

Wavelength [nm]			Spectral irradiance [W · m⁻² · nm⁻¹]
**472**	3.64e-06	3.09e-04	4.80e-05
**473**	3.10e-06	2.82e-04	4.22e-05
**474**	3.08e-06	2.63e-04	4.29e-05
**475**	2.89e-06	2.45e-04	4.19e-05
**476**	2.33e-06	2.32e-04	3.69e-05
**477**	3.05e-06	2.13e-04	3.65e-05
**478**	4.24e-06	1.93e-04	3.81e-05
**479**	3.91e-06	1.83e-04	3.90e-05
**480**	3.75e-06	1.73e-04	3.84e-05
**481**	3.80e-06	1.63e-04	3.59e-05
**482**	3.56e-06	1.53e-04	3.27e-05
**483**	3.29e-06	1.43e-04	2.99e-05
**484**	3.39e-06	1.34e-04	3.10e-05
**485**	3.62e-06	1.26e-04	3.15e-05
**486**	3.97e-06	1.20e-04	3.14e-05
**487**	3.99e-06	1.15e-04	3.25e-05
**488**	4.10e-06	1.10e-04	3.29e-05
**489**	4.76e-06	1.06e-04	3.02e-05
**490**	4.81e-06	1.03e-04	3.35e-05
**491**	4.41e-06	1.00e-04	4.14e-05
**492**	4.31e-06	1.02e-04	4.35e-05
**493**	4.35e-06	1.04e-04	4.53e-05
**494**	4.66e-06	1.07e-04	4.76e-05
**495**	4.27e-06	1.10e-04	4.90e-05
**496**	3.57e-06	1.11e-04	5.02e-05
**497**	4.11e-06	1.12e-04	6.12e-05
**498**	4.48e-06	1.19e-04	7.08e-05
**499**	4.59e-06	1.32e-04	7.79e-05
**500**	4.37e-06	1.41e-04	8.85e-05
**501**	4.21e-06	1.49e-04	9.98e-05
**502**	5.09e-06	1.70e-04	1.08e-04
**503**	5.25e-06	1.89e-04	1.18e-04
**504**	4.68e-06	2.07e-04	1.29e-04
**505**	4.87e-06	2.26e-04	1.42e-04
**506**	4.89e-06	2.48e-04	1.56e-04
**507**	3.94e-06	2.77e-04	1.69e-04
**508**	4.26e-06	3.07e-04	1.89e-04
**509**	5.46e-06	3.38e-04	2.14e-04
**510**	5.37e-06	3.58e-04	2.28e-04
**511**	5.58e-06	3.87e-04	2.46e-04
**512**	6.50e-06	4.31e-04	2.73e-04
**513**	6.36e-06	4.68e-04	2.93e-04
**514**	5.85e-06	5.02e-04	3.12e-04
**515**	6.66e-06	5.38e-04	3.38e-04
**516**	6.65e-06	5.70e-04	3.64e-04
**517**	5.60e-06	5.97e-04	3.89e-04
**518**	6.17e-06	6.17e-04	4.11e-04

Wavelength [nm]			Spectral irradiance [W · m⁻² · nm⁻¹]
**519**	6.90e-06	6.33e-04	4.28e-04
**520**	6.53e-06	6.43e-04	4.27e-04
**521**	6.58e-06	6.60e-04	4.37e-04
**522**	6.98e-06	6.84e-04	4.59e-04
**523**	6.58e-06	6.86e-04	4.70e-04
**524**	6.33e-06	6.86e-04	4.74e-04
**525**	6.61e-06	6.89e-04	4.67e-04
**526**	6.93e-06	6.88e-04	4.68e-04
**527**	7.25e-06	6.86e-04	4.74e-04
**528**	7.31e-06	6.88e-04	4.68e-04
**529**	7.52e-06	6.85e-04	4.59e-04
**530**	7.96e-06	6.73e-04	4.47e-04
**531**	7.50e-06	6.64e-04	4.43e-04
**532**	6.99e-06	6.58e-04	4.40e-04
**533**	7.70e-06	6.53e-04	4.31e-04
**534**	8.22e-06	6.46e-04	4.18e-04
**535**	8.57e-06	6.35e-04	4.03e-04
**536**	8.66e-06	6.14e-04	3.84e-04
**537**	8.79e-06	5.94e-04	3.69e-04
**538**	9.14e-06	5.80e-04	3.63e-04
**539**	9.56e-06	5.66e-04	3.56e-04
**540**	1.00e-05	5.53e-04	3.49e-04
**541**	1.03e-05	5.29e-04	3.36e-04
**542**	1.08e-05	5.04e-04	3.22e-04
**543**	1.17e-05	4.76e-04	3.04e-04
**544**	1.14e-05	4.57e-04	2.97e-04
**545**	1.09e-05	4.41e-04	2.91e-04
**546**	1.11e-05	4.21e-04	2.77e-04
**547**	1.11e-05	4.01e-04	2.61e-04
**548**	1.10e-05	3.81e-04	2.43e-04
**549**	1.02e-05	3.61e-04	2.37e-04
**550**	9.69e-06	3.42e-04	2.32e-04
**551**	9.94e-06	3.21e-04	2.21e-04
**552**	1.02e-05	3.06e-04	2.05e-04
**553**	1.05e-05	2.93e-04	1.86e-04
**554**	1.01e-05	2.78e-04	1.82e-04
**555**	9.82e-06	2.64e-04	1.77e-04
**556**	9.69e-06	2.52e-04	1.71e-04
**557**	9.75e-06	2.39e-04	1.64e-04
**558**	9.87e-06	2.25e-04	1.59e-04
**559**	9.99e-06	2.14e-04	1.58e-04
**560**	1.03e-05	2.06e-04	1.53e-04
**561**	1.07e-05	2.02e-04	1.42e-04
**562**	1.04e-05	1.92e-04	1.34e-04
**563**	1.02e-05	1.84e-04	1.28e-04
**564**	1.07e-05	1.79e-04	1.26e-04
**565**	1.08e-05	1.73e-04	1.25e-04

Wavelength [nm]			Spectral irradiance [W · m⁻² · nm⁻¹]
**566**	1.06e-05	1.66e-04	1.24e-04
**567**	1.08e-05	1.61e-04	1.22e-04
**568**	1.09e-05	1.56e-04	1.18e-04
**569**	1.10e-05	1.50e-04	1.13e-04
**570**	1.11e-05	1.46e-04	1.09e-04
**571**	1.13e-05	1.43e-04	1.07e-04
**572**	1.17e-05	1.39e-04	1.11e-04
**573**	1.19e-05	1.35e-04	1.12e-04
**574**	1.22e-05	1.31e-04	1.11e-04
**575**	1.19e-05	1.34e-04	1.11e-04
**576**	1.17e-05	1.36e-04	1.10e-04
**577**	1.22e-05	1.33e-04	1.12e-04
**578**	1.26e-05	1.31e-04	1.14e-04
**579**	1.30e-05	1.28e-04	1.16e-04
**580**	1.26e-05	1.30e-04	1.24e-04
**581**	1.27e-05	1.36e-04	1.31e-04
**582**	1.34e-05	1.45e-04	1.37e-04
**583**	1.39e-05	1.46e-04	1.46e-04
**584**	1.43e-05	1.46e-04	1.56e-04
**585**	1.46e-05	1.51e-04	1.66e-04
**586**	1.48e-05	1.61e-04	1.79e-04
**587**	1.50e-05	1.74e-04	1.95e-04
**588**	1.54e-05	1.80e-04	2.10e-04
**589**	1.57e-05	1.88e-04	2.26e-04
**590**	1.60e-05	1.99e-04	2.42e-04
**591**	1.64e-05	2.13e-04	2.61e-04
**592**	1.67e-05	2.29e-04	2.82e-04
**593**	1.64e-05	2.52e-04	3.15e-04
**594**	1.63e-05	2.73e-04	3.44e-04
**595**	1.65e-05	2.90e-04	3.69e-04
**596**	1.71e-05	3.15e-04	3.98e-04
**597**	1.76e-05	3.42e-04	4.29e-04
**598**	1.78e-05	3.69e-04	4.68e-04
**599**	1.79e-05	3.97e-04	5.04e-04
**600**	1.80e-05	4.26e-04	5.37e-04
**601**	1.83e-05	4.57e-04	5.72e-04
**602**	1.86e-05	4.89e-04	6.10e-04
**603**	1.92e-05	5.21e-04	6.52e-04
**604**	1.94e-05	5.47e-04	6.86e-04
**605**	1.94e-05	5.72e-04	7.16e-04
**606**	1.98e-05	5.96e-04	7.45e-04
**607**	2.01e-05	6.19e-04	7.72e-04
**608**	2.03e-05	6.41e-04	7.97e-04
**609**	2.11e-05	6.56e-04	8.21e-04
**610**	2.19e-05	6.67e-04	8.44e-04
**611**	2.29e-05	6.70e-04	8.62e-04
**612**	2.29e-05	6.71e-04	8.74e-04

Wavelength [nm]			Spectral irradiance [W · m^{-2} · nm^{-1}]
**613**	2.25e-05	6.71e-04	8.83e-04
**614**	2.26e-05	6.66e-04	8.82e-04
**615**	2.29e-05	6.61e-04	8.81e-04
**616**	2.35e-05	6.56e-04	8.78e-04
**617**	2.32e-05	6.47e-04	8.61e-04
**618**	2.28e-05	6.34e-04	8.42e-04
**619**	2.32e-05	6.16e-04	8.27e-04
**620**	2.38e-05	5.95e-04	8.05e-04
**621**	2.47e-05	5.71e-04	7.77e-04
**622**	2.48e-05	5.63e-04	7.61e-04
**623**	2.48e-05	5.51e-04	7.43e-04
**624**	2.48e-05	5.33e-04	7.23e-04
**625**	2.46e-05	5.17e-04	7.03e-04
**626**	2.44e-05	5.02e-04	6.82e-04
**627**	2.43e-05	4.94e-04	6.61e-04
**628**	2.44e-05	4.80e-04	6.39e-04
**629**	2.48e-05	4.59e-04	6.15e-04
**630**	2.51e-05	4.53e-04	5.94e-04
**631**	2.51e-05	4.46e-04	5.75e-04
**632**	2.46e-05	4.36e-04	5.60e-04
**633**	2.43e-05	4.23e-04	5.42e-04
**634**	2.40e-05	4.11e-04	5.23e-04
**635**	2.38e-05	4.06e-04	5.05e-04
**636**	2.41e-05	4.00e-04	4.88e-04
**637**	2.53e-05	3.93e-04	4.69e-04
**638**	2.50e-05	3.81e-04	4.57e-04
**639**	2.46e-05	3.68e-04	4.45e-04
**640**	2.47e-05	3.54e-04	4.30e-04
**641**	2.48e-05	3.43e-04	4.17e-04
**642**	2.48e-05	3.36e-04	4.05e-04
**643**	2.44e-05	3.26e-04	3.87e-04
**644**	2.39e-05	3.14e-04	3.72e-04
**645**	2.31e-05	2.99e-04	3.62e-04
**646**	2.39e-05	2.95e-04	3.48e-04
**647**	2.46e-05	2.90e-04	3.35e-04
**648**	2.39e-05	2.78e-04	3.28e-04
**649**	2.37e-05	2.65e-04	3.17e-04
**650**	2.38e-05	2.52e-04	3.04e-04
**651**	2.40e-05	2.46e-04	2.94e-04
**652**	2.40e-05	2.38e-04	2.84e-04
**653**	2.37e-05	2.25e-04	2.75e-04
**654**	2.34e-05	2.23e-04	2.70e-04
**655**	2.32e-05	2.22e-04	2.64e-04
**656**	2.28e-05	2.12e-04	2.51e-04
**657**	2.26e-05	2.04e-04	2.43e-04
**658**	2.26e-05	1.98e-04	2.38e-04
**659**	2.33e-05	1.90e-04	2.30e-04

Wavelength [nm]			Spectral irradiance [W · m^{-2} · nm^{-1}]
**660**	2.38e-05	1.82e-04	2.22e-04
**661**	2.39e-05	1.73e-04	2.12e-04
**662**	2.35e-05	1.66e-04	2.05e-04
**663**	2.31e-05	1.59e-04	1.97e-04
**664**	2.40e-05	1.52e-04	1.83e-04
**665**	2.39e-05	1.46e-04	1.78e-04
**666**	2.31e-05	1.43e-04	1.80e-04
**667**	2.34e-05	1.38e-04	1.77e-04
**668**	2.34e-05	1.34e-04	1.72e-04
**669**	2.29e-05	1.30e-04	1.67e-04
**670**	2.32e-05	1.23e-04	1.59e-04
**671**	2.37e-05	1.17e-04	1.50e-04
**672**	2.33e-05	1.21e-04	1.47e-04
**673**	2.32e-05	1.20e-04	1.45e-04
**674**	2.34e-05	1.14e-04	1.44e-04
**675**	2.36e-05	1.12e-04	1.35e-04
**676**	2.36e-05	1.10e-04	1.30e-04
**677**	2.33e-05	1.06e-04	1.32e-04
**678**	2.34e-05	1.06e-04	1.28e-04
**679**	2.37e-05	1.05e-04	1.23e-04
**680**	2.36e-05	9.88e-05	1.15e-04
**681**	2.35e-05	9.38e-05	1.11e-04
**682**	2.34e-05	9.03e-05	1.11e-04
**683**	2.37e-05	9.23e-05	1.13e-04
**684**	2.40e-05	9.43e-05	1.12e-04
**685**	2.41e-05	9.51e-05	1.07e-04
**686**	2.35e-05	9.20e-05	1.04e-04
**687**	2.29e-05	8.77e-05	1.01e-04
**688**	2.33e-05	8.61e-05	9.69e-05
**689**	2.35e-05	8.23e-05	9.15e-05
**690**	2.35e-05	7.66e-05	8.55e-05
**691**	2.34e-05	7.67e-05	9.00e-05
**692**	2.31e-05	7.69e-05	9.16e-05
**693**	2.26e-05	7.66e-05	8.54e-05
**694**	2.29e-05	7.39e-05	8.14e-05
**695**	2.33e-05	7.10e-05	7.80e-05
**696**	2.25e-05	7.27e-05	7.34e-05
**697**	2.21e-05	7.13e-05	7.20e-05
**698**	2.21e-05	6.73e-05	7.34e-05
**699**	2.18e-05	6.45e-05	7.49e-05
**700**	2.17e-05	6.19e-05	7.50e-05
**701**	2.19e-05	5.94e-05	7.27e-05
**702**	2.15e-05	5.84e-05	6.68e-05
**703**	2.10e-05	5.78e-05	6.09e-05
**704**	2.10e-05	5.72e-05	6.05e-05
**705**	2.08e-05	5.68e-05	6.11e-05
**706**	2.06e-05	5.64e-05	6.25e-05

Wavelength [nm]			Spectral irradiance [W · m⁻² · nm⁻¹]
**707**	2.06e-05	5.12e-05	6.16e-05
**708**	2.04e-05	4.75e-05	5.94e-05
**709**	2.00e-05	4.66e-05	5.53e-05
**710**	1.99e-05	4.68e-05	5.38e-05
**711**	1.96e-05	4.77e-05	5.30e-05
**712**	1.87e-05	5.02e-05	5.16e-05
**713**	1.83e-05	4.80e-05	4.97e-05
**714**	1.82e-05	4.25e-05	4.75e-05
**715**	1.77e-05	3.97e-05	4.50e-05
**716**	1.73e-05	3.86e-05	4.24e-05
**717**	1.69e-05	4.00e-05	3.98e-05
**718**	1.65e-05	3.87e-05	3.88e-05
**719**	1.61e-05	3.74e-05	3.85e-05
**720**	1.56e-05	3.91e-05	3.90e-05
**721**	1.54e-05	3.65e-05	3.88e-05
**722**	1.54e-05	3.12e-05	3.81e-05
**723**	1.49e-05	2.98e-05	3.24e-05
**724**	1.45e-05	2.92e-05	3.03e-05
**725**	1.42e-05	2.94e-05	3.33e-05
**726**	1.38e-05	3.10e-05	3.43e-05
**727**	1.33e-05	3.13e-05	3.40e-05
**728**	1.29e-05	2.66e-05	3.07e-05
**729**	1.27e-05	2.49e-05	2.90e-05
**730**	1.27e-05	2.48e-05	2.85e-05
**731**	1.25e-05	2.40e-05	3.11e-05
**732**	1.19e-05	2.31e-05	3.05e-05
**733**	1.11e-05	2.19e-05	2.60e-05
**734**	1.10e-05	2.27e-05	2.66e-05
**735**	1.10e-05	2.37e-05	2.72e-05
**736**	1.10e-05	2.44e-05	2.55e-05
**737**	1.04e-05	2.30e-05	2.45e-05
**738**	9.69e-06	2.05e-05	2.37e-05
**739**	9.96e-06	1.74e-05	2.01e-05
**740**	9.80e-06	1.72e-05	1.80e-05
**741**	9.20e-06	1.98e-05	1.73e-05
**742**	9.18e-06	1.91e-05	1.45e-05
**743**	8.96e-06	1.88e-05	1.45e-05
**744**	8.26e-06	2.00e-05	2.04e-05
**745**	8.45e-06	1.73e-05	2.03e-05
**746**	8.74e-06	1.44e-05	1.85e-05
**747**	7.97e-06	1.65e-05	1.77e-05
**748**	7.78e-06	1.57e-05	1.78e-05
**749**	7.99e-06	1.29e-05	1.85e-05
**750**	7.51e-06	1.41e-05	1.36e-05
**751**	7.28e-06	1.50e-05	1.16e-05
**752**	7.41e-06	1.56e-05	1.37e-05
**753**	7.17e-06	1.40e-05	1.46e-05

Wavelength [nm]			Spectral irradiance [W · m⁻² · nm⁻¹]
**754**	6.78e-06	1.28e-05	1.44e-05
**755**	6.22e-06	1.38e-05	1.13e-05
**756**	5.95e-06	1.39e-05	1.15e-05
**757**	5.83e-06	1.36e-05	1.34e-05
**758**	5.80e-06	1.21e-05	1.59e-05
**759**	5.29e-06	1.10e-05	1.60e-05
**760**	4.34e-06	1.04e-05	1.38e-05
**761**	5.26e-06	1.34e-05	9.99e-06
**762**	5.78e-06	1.43e-05	7.67e-06
**763**	5.41e-06	1.10e-05	8.25e-06
**764**	5.54e-06	1.16e-05	7.74e-06
**765**	5.67e-06	1.15e-05	7.13e-06
**766**	5.29e-06	5.51e-06	7.12e-06
**767**	4.59e-06	4.23e-06	8.23e-06
**768**	3.88e-06	5.62e-06	9.95e-06
**769**	4.17e-06	1.01e-05	1.21e-05
**770**	4.76e-06	1.26e-05	8.29e-06
**771**	5.55e-06	1.36e-05	1.80e-07
**772**	4.98e-06	8.36e-06	4.56e-06
**773**	4.58e-06	5.42e-06	6.58e-06
**774**	4.35e-06	5.13e-06	5.87e-06
**775**	4.21e-06	5.11e-06	7.91e-06
**776**	4.09e-06	4.49e-06	8.95e-06
**777**	4.00e-06	2.51e-06	7.13e-06
**778**	4.06e-06	8.05e-06	6.65e-06
**779**	4.19e-06	1.39e-05	6.24e-06
**780**	4.45e-06	8.88e-06	4.24e-06

Step 6. Share an online version of this report.

https://luox.app/u/spd1,380,1,wi,-61,AAAAAAAAAAAAAAAAAAAAAAAAAAAAAAAAAAAADxEYCOAAAAAAAAAAJ5PrNXNMRUQtNqM

Click above to copy the URL to your clipboard
