# Supplementary material for: Preliminary Results: The Impact of Smartphone Use and Short-Wavelength Light during the Evening on Circadian Rhythm, Sleep and Alertness
Source: Clocks Sleep. 2021 Jan 22;3(1):66–86. doi: 10.3390/clockssleep3010005 (PMC7838958; doi:10.3390/clockssleep3010005)
Supplement: Supplementary file 1 [file clockssleep-03-00005-s001.zip › Table_S1.pdf]

Table S2. Mean subjective ratings and standard deviations for the properties of the room lighting and smartphone screen.

Room Lighting

	**Book**	**No Filter**	**Filter**	***p*-value (η_p^2)**
Bright	3.71 (1.94)	4.07 (1.59)	3.64 (1.33)	.650 (.03)
Pleasant	7.14 (2.11)	6.64 (1.82)	6.86 (1.75)	.527 (.05)
Activating	3.79 (1.85)	4.00 (1.62)	3.71 (2.13)	.852 (.01)
Dazzling	1.00 (1.24)	1.64 (1.78)	1.07 (1.21)	.224 (.11)
Warm	5.64 (2.10)	6.21 (1.89)	6.07 (1.59)	.602 (.04)

Smartphone Screen

	**No Filter**	**Filter**	***p*-value (*d*)**
Bright	7.57 (1.50)	5.21 (1.76)	.003 (0.97)
Pleasant	3.64 (2.27)	6.14 (2.18)	.009 (-0.82)
Activating	5.79 (2.75)	4.64 (1.45)	.108 (0.46)
Dazzling	6.43 (3.11)	3.71 (2.49)	$.003^{**}$ (0.99)
Warm	3.00 (1.24)	6.29 (2.23)	<.001 (-1.47)

Note. Participants (*N* = 14) rated how each adjective described the two light sources on a scale from 0 (not at all) to 10 (very).
