# Supplementary figures and images for: Preliminary Results: The Impact of Smartphone Use and Short-Wavelength Light during the Evening on Circadian Rhythm, Sleep and Alertness
Source: Clocks Sleep. 2021 Jan 22;3(1):66–86. doi: 10.3390/clockssleep3010005 (PMC7838958; doi:10.3390/clockssleep3010005)

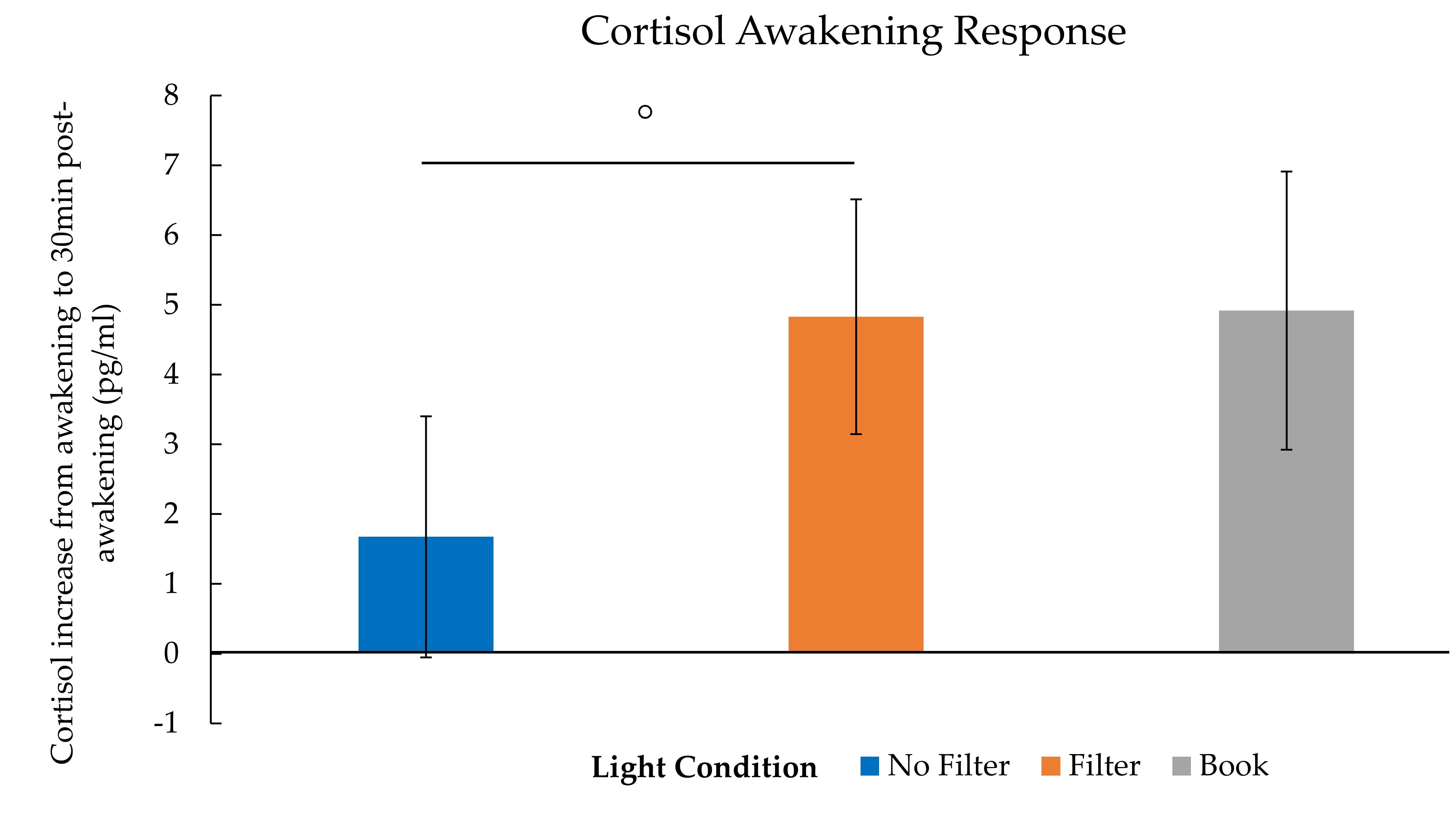

Supplement: Supplementary file 1 [file clockssleep-03-00005-s001.zip › Figure_S1.jpg]

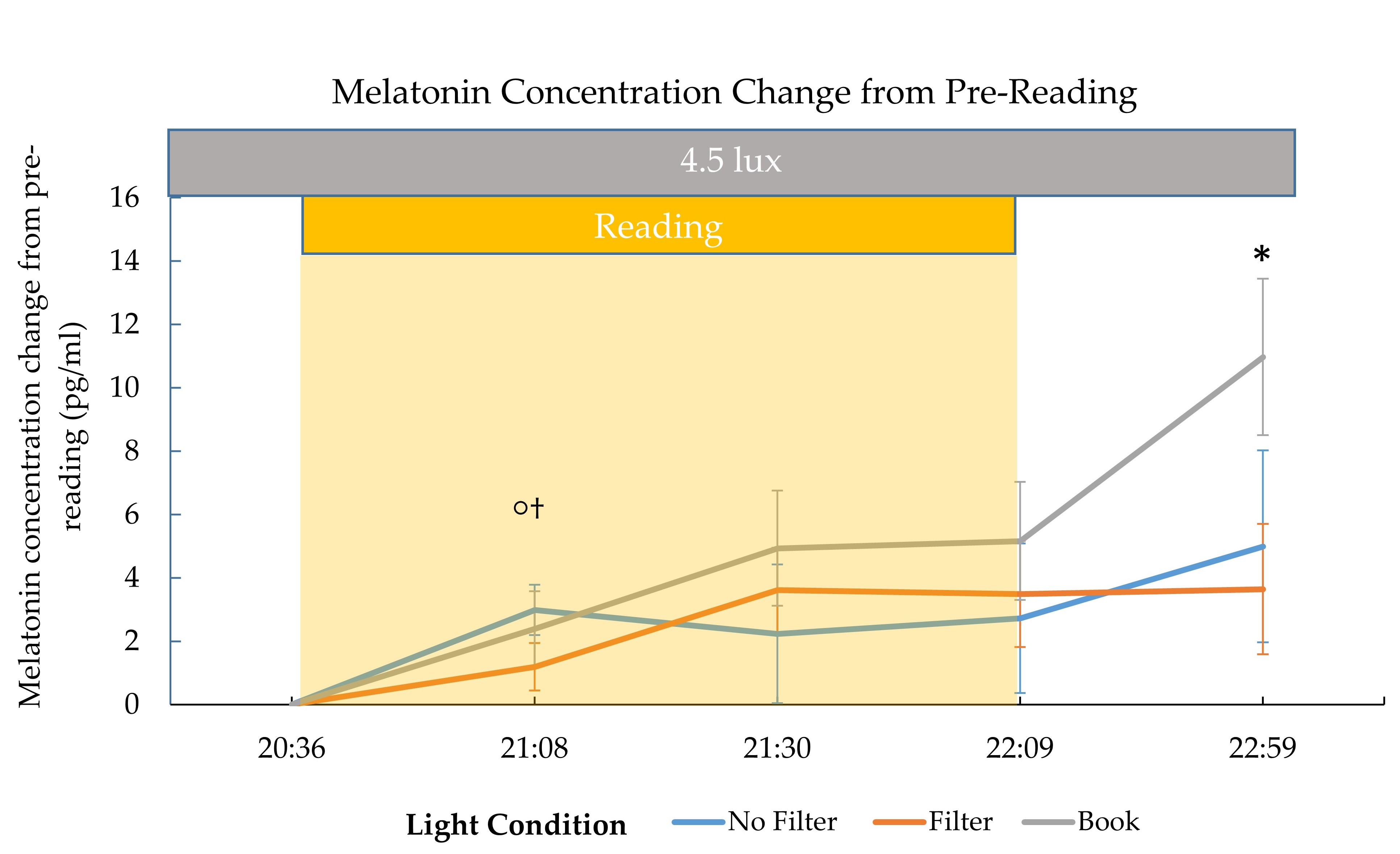

Supplement: Supplementary file 1 [file clockssleep-03-00005-s001.zip › Figure_S2.jpg]

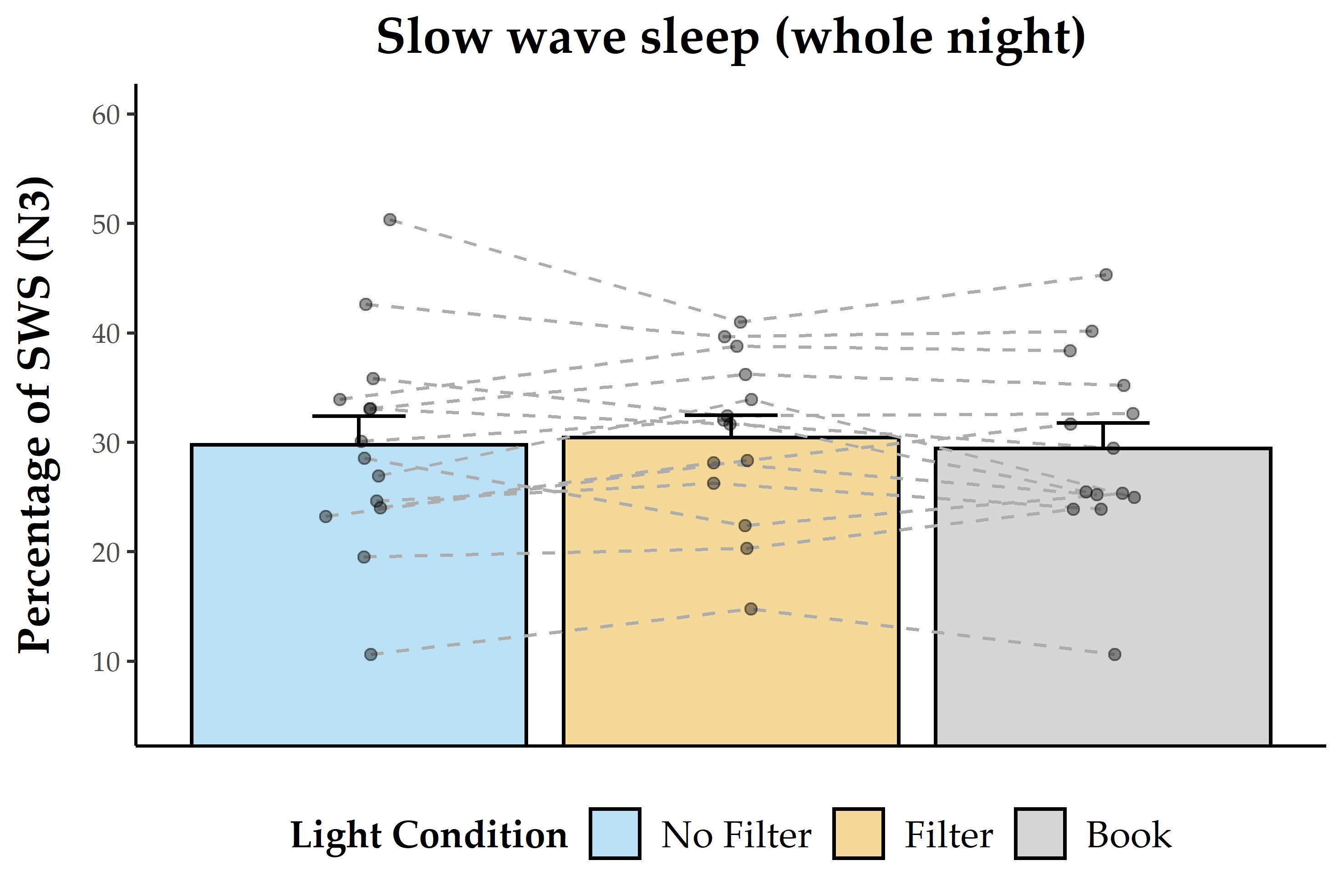

Supplement: Supplementary file 1 [file clockssleep-03-00005-s001.zip › Figure_S3.jpeg]

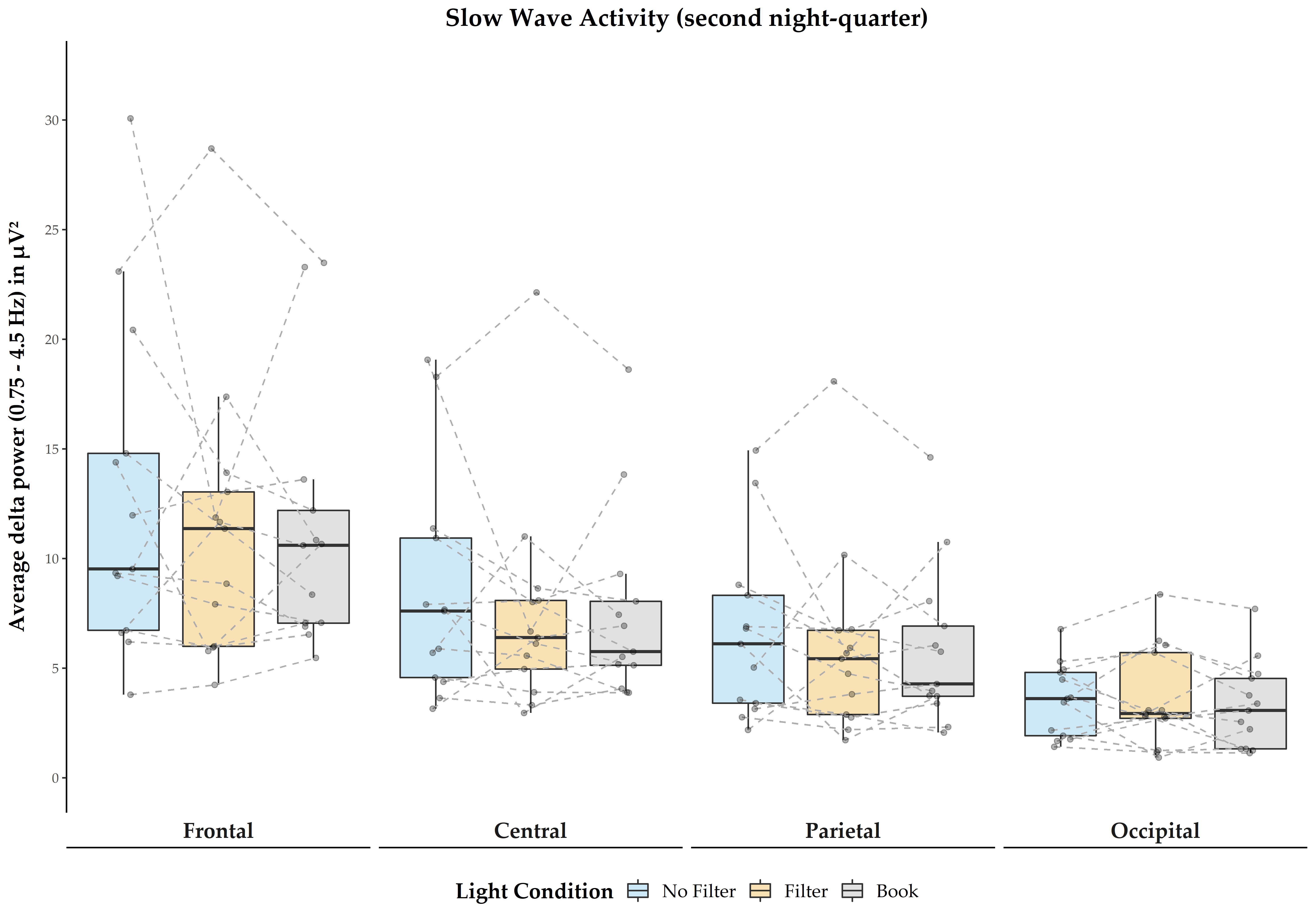

Supplement: Supplementary file 1 [file clockssleep-03-00005-s001.zip › Figure_S4.jpeg]

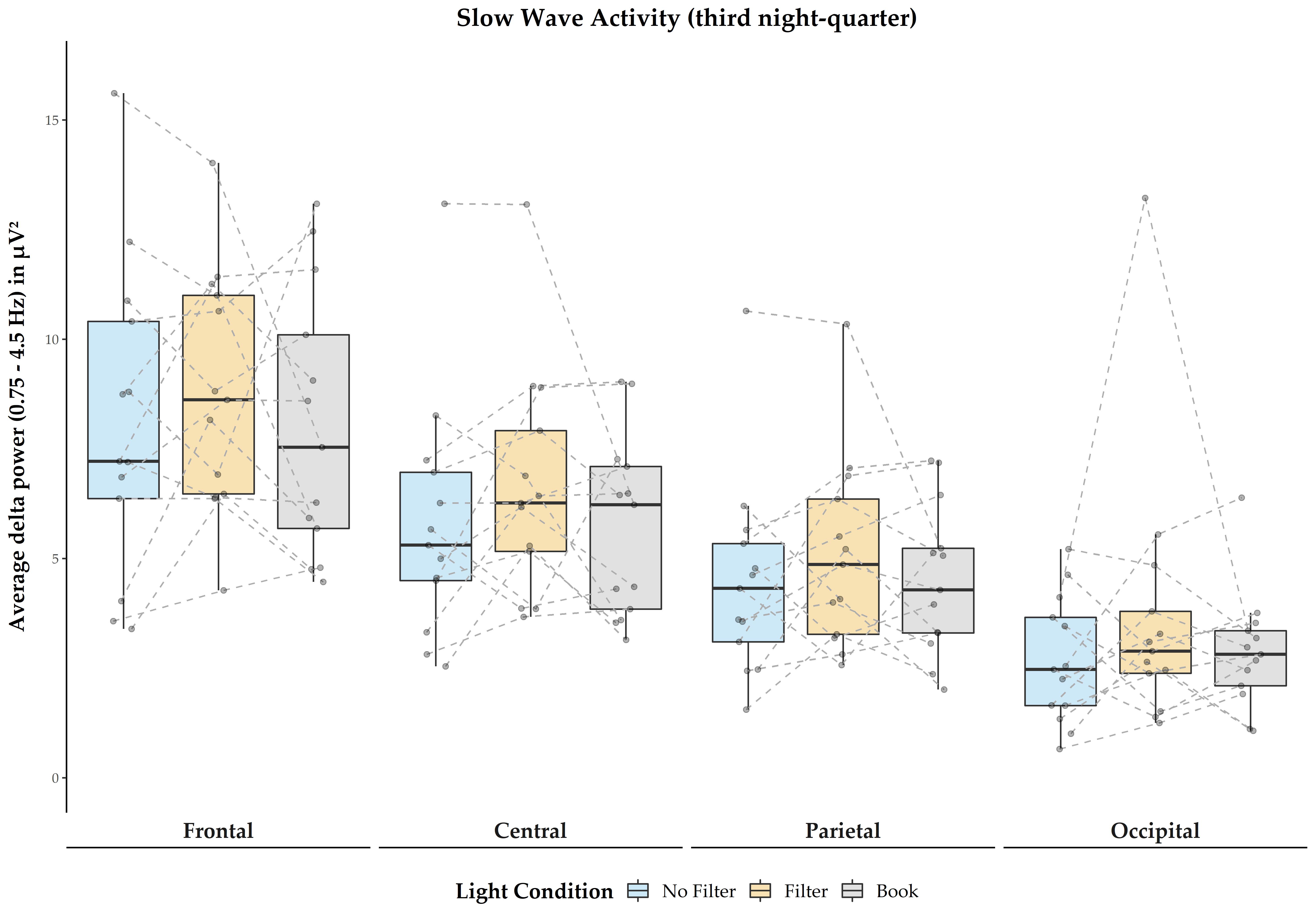

Supplement: Supplementary file 1 [file clockssleep-03-00005-s001.zip › Figure_S5.jpeg]

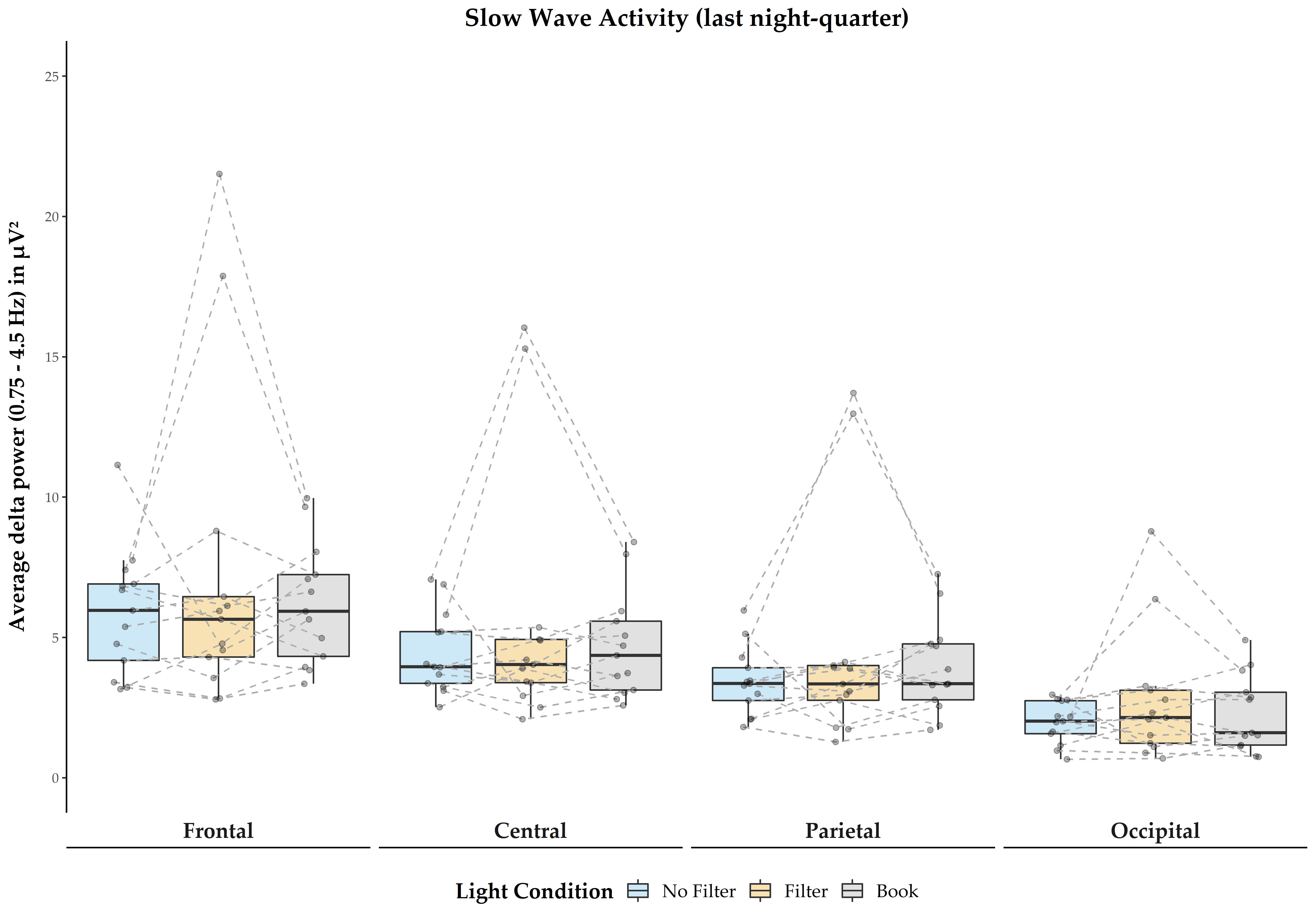

Supplement: Supplementary file 1 [file clockssleep-03-00005-s001.zip › Figure_S6.jpeg]

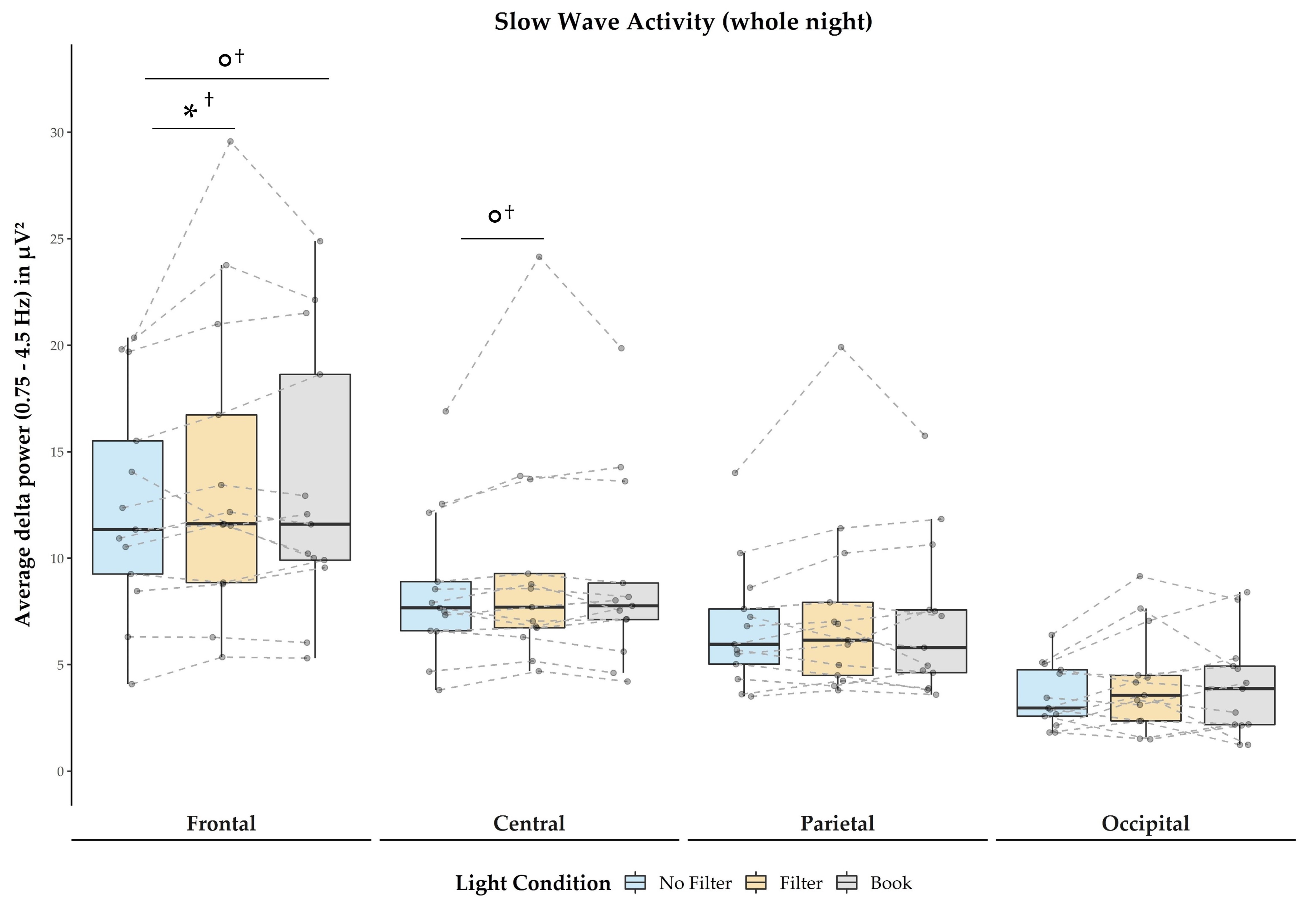

Supplement: Supplementary file 1 [file clockssleep-03-00005-s001.zip › Figure_S7.jpg]

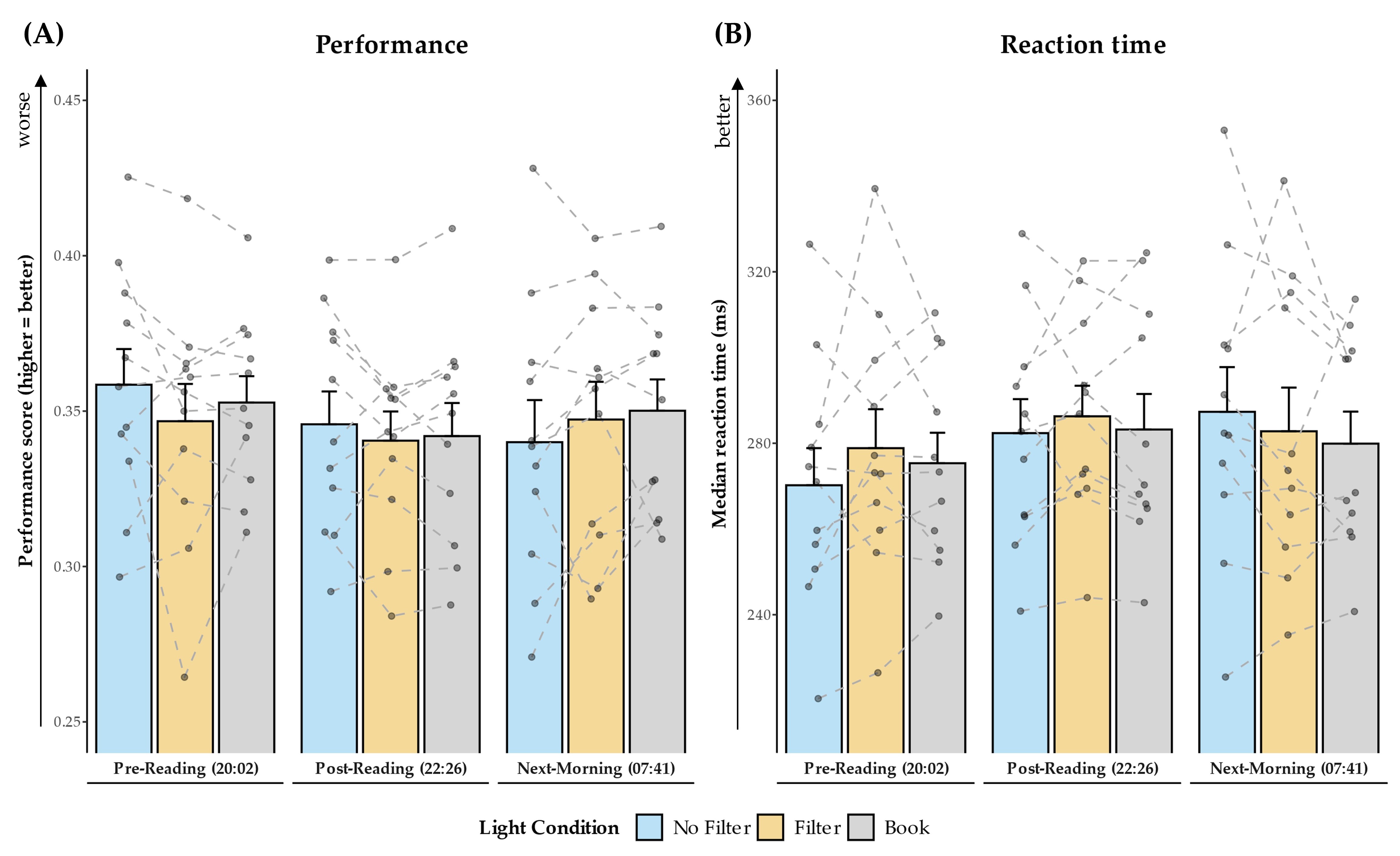

Supplement: Supplementary file 1 [file clockssleep-03-00005-s001.zip › Figure_S8.jpg]
